# Quantification of Dynamic Morphological Drug Responses in 3D Organotypic Cell Cultures by Automated Image Analysis

**DOI:** 10.1371/journal.pone.0096426

**Published:** 2014-05-08

**Authors:** Ville Härmä, Hannu-Pekka Schukov, Antti Happonen, Ilmari Ahonen, Johannes Virtanen, Harri Siitari, Malin Åkerfelt, Jyrki Lötjönen, Matthias Nees

**Affiliations:** 1 Medical Biotechnology Knowledge Centre, VTT Technical Research Centre of Finland, Turku, Finland; 2 Turku Centre for Biotechnology, University of Turku, Turku, Finland; 3 Department of Signal Processing, Tampere University of Technology, Tampere, Finland; 4 Department of Information Technology, University of Turku, Turku, Finland; 5 Knowledge Intensive Services, VTT Technical Research Centre of Finland, Tampere, Finland; Ghent University, Belgium

## Abstract

Glandular epithelial cells differentiate into complex multicellular or acinar structures, when embedded in three-dimensional (3D) extracellular matrix. The spectrum of different multicellular morphologies formed in 3D is a sensitive indicator for the differentiation potential of normal, non-transformed cells compared to different stages of malignant progression. In addition, single cells or cell aggregates may actively invade the matrix, utilizing epithelial, mesenchymal or mixed modes of motility. Dynamic phenotypic changes involved in 3D tumor cell invasion are sensitive to specific small-molecule inhibitors that target the actin cytoskeleton. We have used a panel of inhibitors to demonstrate the power of automated image analysis as a phenotypic or morphometric readout in cell-based assays. We introduce a streamlined stand-alone software solution that supports large-scale high-content screens, based on complex and organotypic cultures. AMIDA (**A**utomated **M**orphometric **I**mage **D**ata **A**nalysis) allows quantitative measurements of large numbers of images and structures, with a multitude of different spheroid shapes, sizes, and textures. AMIDA supports an automated workflow, and can be combined with quality control and statistical tools for data interpretation and visualization. We have used a representative panel of 12 prostate and breast cancer lines that display a broad spectrum of different spheroid morphologies and modes of invasion, challenged by a library of 19 direct or indirect modulators of the actin cytoskeleton which induce systematic changes in spheroid morphology and differentiation versus invasion. These results were independently validated by 2D proliferation, apoptosis and cell motility assays. We identified three drugs that primarily attenuated the invasion and formation of invasive processes in 3D, without affecting proliferation or apoptosis. Two of these compounds block Rac signalling, one affects cellular cAMP/cGMP accumulation. Our approach supports the growing needs for user-friendly, straightforward solutions that facilitate large-scale, cell-based 3D assays in basic research, drug discovery, and target validation.

## Introduction

Using a combination of cell culture, microscopic and live-cell imaging techniques, cell lines or primary cells from patients, grown in 3D matrices, can be used to investigate key mechanisms inherent to cancer biology. 3D models are increasingly considered more biologically relevant than 2D monolayer cultures on plastic. However, only recently, the first steps towards systematic characterization and standardization efforts have been undertaken, e.g. by correlating 2D and 3D growth conditions with multicellular morphology, phenotype and molecular signalling [1]. A series of recent reviews demonstrate the growing interest in the technicalities [2], [3] and cell biology [4], [5] of standardized 3D cultures. 3D models are further suitable to address complex aspects of normal and malignant tissues, such as the extracellular matrix (ECM), basement membrane (BM), cell-cell and cell-matrix adhesion, tumour-stroma interactions, cell motility and the formation of relevant tumor-like histology. Mimicking the tumour microenvironment (TME) is further deemed important for modelling long-term drug responses, therapy failure, local invasion and metastasis, and resistance formation.

We and others have demonstrated that biologically relevant, miniaturised 3D models can be cost-effective, robust, reproducible, and fully standardised [6], [7]. Integrated 3D platforms are beginning to enable sufficient throughput for high-content screening (HCS) in both academia and pharmaceutical industry. The increasing availability of primary, patient-derived cell culture materials [8], [9] will further increase their relevance in future. However, a broad biological consensus and general acceptance for experimental 3D platforms is still missing. In particular, it remains unclear which models may be most representative and faithfully recapitulate which aspect(s) of tumour biology. The broad spectrum of 3D models includes spheroid culture in non-adherent conditions, devoid of any biologically relevant matrices, e.g. by using hanging-drop plates [10]; magnetic levitation [11] or stirred bioreactors [12], or when embedded into chemically inert scaffolds (e.g. soft-agar, alginate, or methyl-cellulose). Spheres of tumor cells forming in these settings [13]–[15] are often enriched in stem- and progenitor-like cells, display increased self-renewal potential, but typically fail to develop epithelial characteristics such as a acinar morphogenesis, a functional BM or a hollow lumen. Standardized variants of these basic principles have recently gained attention as a method for propagation of primary (tumour) cells [16], [17]; further enhanced by the use of small-molecule inhibitors or ligands and growth factors that promote stem- and progenitor cell propagation. [18], [19]. In contrast, approaches that utilize re-aggregation of primary tissue cultures into functional 3D matrices or scaffolds can lead to the formation of complex, functional organoids or microtissues that naturally include stromal and ECM components [20]. The direct embedding of cell lines, primary cells [8], [9] or primary explants [21], [22] into biological relevant ECM preparations remains the most promising and practical method to recapitulate morphologic aspects such as tissue formation, differentiation and homeostasis; also including tumor progression and invasion (reviewed in [4]). In addition, it is critical to assess the physical force, pressure and local stiffness or rigidity of the matrix, which promotes tumor progression, cell motility and impacts on the modes of cell invasion used by cancer cells [23], [24]. The strongest differentiation-inducing effects on cells of epithelial origin are typically observed with laminin-rich BM extracts such as Matrigel [25]. These promote maturation and apico-basal polarity of multicellular epithelial structures [26], including cell-cell and cell-matrix contacts [27].

The differentiation potential of malignant glandular cancer cells, compared to normal, non-transformed epithelial cells is typically compromised by oncogenic mutations, activation of growth-promoting, and differentiation-blocking signalling pathways (e.g. PI3Kinase, AKT, mTOR and c-src pathways [28], reviewed in [29], [30]. Accordingly, morphologies formed in 3D range from well-polarized acini with complete BM and a hollow lumen, to “round” spheres lacking either of these properties, eventually forming increasingly irregular “grape-like” or “stellate” cell masses by gradually losing cell-cell adhesion [31], [32]. Thus, not only tumor cells, but also multicellular tumor spheroids can display striking morphologic plasticity [33], [34]. The most advanced progression stages are related to overt invasion into the surrounding ECM. These various spheroid or acinus phenotypes correlate with incremental activation of oncogenic signalling pathways and re-arrangement of the cytoskeleton in tumor progression [34], [35]. Imaging-based analyses of 3D morphology can therefore be highly informative for *in vitro* tumour biology, based on cancer cell lines [36], [37] or primary, patient-derived tissue cultures [21], [38]. This approach can be further assisted by mathematical modelling [39]–[41], machine learning and Bayesian networks [42].

Advanced morphological image analysis tools are already widely established for 2D cultures, but only emerging in the 3D field. *ImageJ* represents the most widely used open-source image analysis software. Both *CellC* [43], [44] and *CellProfiler* [45], [46] are open-source software programs, specifically tailored for high-content analyses of microscopic images of (mainly) single cells. None of these applications have been specifically developed, nor optimized for organotypic 3D cultures, and do not support analysis of image sets on a large-scale. Morphologies formed in 3D vary in shape, size, geometry, density, surface features, and internal textures. In addition, quantitating the characteristic dynamic changes observed in 3D cultures (invasion, differentiation and de-differentiation) requires specific and accurate measurement of the most informative and biologically relevant morphometric features. Existing image analysis programs currently do not handle large numbers of 3D objects that need to be segmented, and further processing of the resulting numerical data for subsequent, statistically relevant mathematical analyses. This approach requires the balance between high content, typically on the expense of experimental throughput. Ideally, a suitable strategy to speed up experimental throughput should focus primarily on multicellular and tissue-related features, instead of detailed single-cell analyses. In small-scale (i.e. most basic research) studies, it is possible to perform image segmentation in semi-automated fashion (such as in [47]), and programs such as CellProfiler or Volocity may be ideally suited for this purpose. However, in miniaturized 3D tissue platforms with larger numbers of compounds or other perturbants, the analysis of complexity and heterogeneity develops rapidly beyond human sensorial capacity. Even visual inspection of large 3D image series, e.g. for quality control purposes, becomes laborious, time consuming, and subject to human error. Fully automated morphometric or phenotypic image analysis solutions are required to measure multiple levels of structural information in a reproducible, accurate, unbiased and quantitative way. Since structures formed in 3D are complex and heterogeneous, any representative quantitation of multicellular structures will benefit from applying a large panel of geometrical image processing criteria, in parallel or subsequently. Ideally, these are based on mathematic algorithms that match the complex nature of the structures analysed. With this open approach, quantitative image analysis is capable to detect multiple levels of overt (visible) as well as hidden phenotypic changes, undetectable to the naked eye [48]. To warrant significant throughput, reduced cost and a high level of miniaturization and standardization, suitable 3D platforms and matching 3D image analysis tools must be fully compatible with each other and existing laboratory automation. They must further comply with cell culture standards, HCS instrumentation and microscopes, robotics (liquid handling), plate readers etc. However, no satisfactory, fully integrated and sufficiently user-friendly solution that addresses all of these aspects simultaneously exists to date.

This manuscript describes a combined approach, illustrating how 3D cultures can be generated in miniaturized and standardized fashion, and subsequently analysed by a dedicated software package that specifically and quantitatively addresses the complex phenotypes formed under these conditions.

## Materials and Methods

### Cell lines and culture conditions

All cell lines were obtained from American Type Culture Collection (Manassas, VA, USA) or originator laboratories. Table S1 lists all cell lines used in this manuscript. MDA-MB-231 SA cells were a kind gift from Therese Guise, Indiana University, Indianapolis, IN, USA. PrCa lines were propagated in RPMI-1640 (Sigma-Aldrich, St. Louis, MO, USA), BrCa lines in DMEM (Sigma-Aldrich, St. Louis, MO, USA) supplemented with 10% FBS, 1% penicillin/streptomycin and 1% L-glutamine. Immortalized, non-transformed epithelial cell lines EP156T and RWPE-1 cells were cultured in Keratinocyte Serum-Free Medium (KSFM; Invitrogen, Carlsbad, CA, USA), supplemented with 50 mg/l bovine pituitary extract, 5 mg/l EGF and 2% FBS for 3D conditions. 1 nM R1881 was added to LAPC-4 medium for growth support.

### Miniaturized 3D cell cultures

All of the experiments shown were performed in low throughput 15-well Angiogenesis slides (Ibidi GmbH, Munich, Germany), and growth factor-reduced Matrigel (BD Invitrogen) as the ECM of choice to promote differentiation. Miniaturized 3D cultures were prepared as described previously [6], [7]. Bottom wells of ibidi Angiogenesis μ-slides were filled with 10 µl of 50% Matrigel-medium (typically 3–5 mg/ml protein, depending on the batch), and incubated at +37°C for 30–60 min. Cells were placed on top of the polymerised bottom gel at a density of 700–1500 cells/well (depending on the cell line), and incubated at +37°C for 1–2 h. Medium was discarded, and cell layers covered with 20 µl of 25% Matrigel (1.5–2.5 mg/ml depending on the batch). The μ-slides were humidified by adding 15 µl drops of sterile water between the wells. The upper gel was allowed to polymerize at +37°C for 3–4 h or overnight. Wells were then filled with medium, and changed every second day.

### Compound treatments

All compounds were purchased from Tocris (Bristol, UK), except for Ki-16425 (Cayman Chemical Company, Ann Arbor, MI, USA) and CCG-1423, Rac inhibitor I (#553502) and Rac inhibitor II (#553511) (Merck Millipore, Darmstadt, Germany), and dissolved in appropriate vehicle (DMSO, EtOH or PBS). All drug exposures were performed in triplicates, including vehicle (DMSO) controls. Compound treatments were initiated after 4 days of 3D culture, and continued for an additional 6 days, after which spheroids were stained and imaged. For two cell lines, PC-3M and ALVA31, which showed a strongly invasive phenotype, drug treatments were initiated already on day two, and emerging structures imaged on day 8–9. More slowly growing LAPC-4 and EP156T spheroids were incubated for 8 days prior to treatments.

### 3D image acquisition, pre-processing, and morphometric analyses

Multicellular structures were double-stained with SYTO 62 fluorescent dye (Invitrogen) and NucView caspase-3 detection reagent (Essen Bioscience). 3D confocal images were acquired with a Zeiss Axiovert-200M microscope, equipped with Yokogawa CSU22 spinning disc confocal unit using Zeiss Plan-Neofluar 5x objective. Intensity projections were created with SlideBook (Intelligent Imaging Innovations Inc, Denver, CO, USA). Background noise was removed by normalization, using either SlideBook or ImageJ (NIH, Bethesda, MD, USA) programs. The AMIDA program can be freely downloaded and is also available as supplementary file (AMIDA Program S1). Also a collection of exemplary images used for analyses performed by AMIDA, as shown in this manuscript, is available as a supplementary data file (Image Data S1).

### 2D cell migration and invasion assays (CellPlayer)

For invasion assays, 96-well ImageLock plates (Essen Bioscience, Ann Arbor, MI, USA) were pre-coated overnight at +4°C with 10% Growth factor-reduced (GFR) Matrigel. In both assays, cells were grown to confluence on ImageLock plates, and wounded with the WoundMaker device (Essen Bioscience). Detached cells were removed by aspiration. For cell migration assays, the compounds were mixed with cell culture medium. For cell invasion assays, compounds were mixed into medium and 25% GFR-Matrigel;fresh medium was added after 48 h. Wound closure was monitored and quantified with the IncuCyte live-cell imager (Essen Bioscience).

### 2D proliferation assay

Cells were transferred into 384-well plates (Corning, New York, NY, USA) at a density of 1500 cells/well and incubated overnight at +37°C. The compounds were mixed into medium and pipetted into the wells. Plates were incubated at +37°C for 72 h, and nuclei subsequently stained with Hoechst DNA dye. The number of nuclei was measured with Olympus ScanR microscope (Olympus, Shinjuku, Tokyo, Japan).

### 2D apoptosis assay

Cells were transferred into 96-well plates (Corning) at a density of 8000 cells/well, and incubated overnight at +37°C. The compounds were mixed in the culture medium and pipetted into wells together with 3.3 µM DEVD-NucView kinetic caspase-3/7 reagent (Essen Bioscience), incubated at +37°C for 72 h, and monitored in real time with an IncuCyte FLR fluorescent imaging device (Essen Bioscience). Confluency and number of apoptotic cells per image were quantified by IncuCyte software (version 2011A).

### Data annotation, quality control and statistical analyses

All statistical analysis and plotting tools implemented for processing numerical data (post-image analysis) were written by an expert statistician using R, an open source programming language and software environment for statistical computing and graphics (http://cran.r-project.org). All R scripts were incorporated in REX, an in-house html-software environment that includes a browser-based user interface.

Heatmaps display the difference in medians between the treatments and the control for the selected features. The calculated median differences are standardized to have unit variance, in order to account for the varying scales in features. The statistical significance of the observed differences in medians is assessed using the nonparametric Mann-Whitney U-test. The obtained p-values are then Bonferroni-corrected, multiplied by the number of treatments. When necessary, heatmaps have been clustered using complete linkage of Euclidean distances. Time series analyses were done as described in [60].

## Results

### 3D cell culture platform and cell culture models

**Figure 1:**
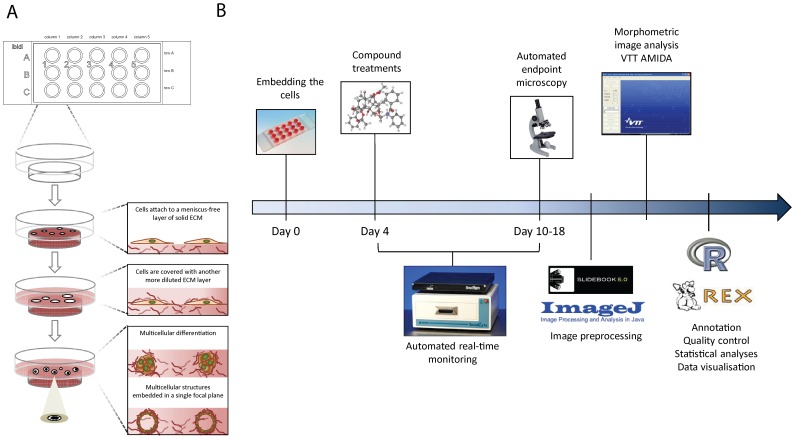
Our compound screening concept is based on a simple cell culture platform optimised for 3D spheroid cultures complemented with an easy to use proprietary image analysis program and data analysis tools. (A) ibidi Angiogenesis μ-slides and μ-plates have a unique well-in-a-well design that facilitates 3D cell culturing between two layers of extracellular matrix on a very narrow focal plane. (B) Time and operation schedule for a typical compound screen including all the major steps from cell seeding to image analysis and visualisation of morphometric responses.

Our cell culture platform is based on two ibidi Angiogenesis products, namely the Angiogenesis 15-well μ-slides or the larger 96-well μ-plates (Ibidi GmbH, Munich, Germany), both featuring an identical well-in-a-well geometry that consists of two compartments, the smaller residing within the bottom of the larger well. This geometry reduces the curvature or meniscus caused by liquid tension, resulting in even liquid surface. This allows cells to be embedded in a defined and narrow focal plane embedded between two layers of ECM (Figure 1A). As ECM, synthetic hydrogels, alginates, soft agar and methyl-cellulose, or biologically relevant matrices such as collagen type I and Matrigel can be used. Over 30 prostate cancer (PrCa) cell lines, derivatives and primary prostate cells have been tested with this standardized 3D cell culture platform [6], [7]. Most tumour lines form either round, irregular (mass), or invasive (stellate) multicellular spheroids. One of the most interesting and heterogeneous tumor lines is PC-3, characterized by extreme epithelial plasticity: PC-3 spheroids initially form well-differentiated, polarized and hollow acinar spheroids. After 6–8 days, these structures spontaneously revert to rapidly invasive multicellular or string-like structures, indicating a mesenchymal mode of cell motility. PC-3 cells represent a particularly suitable model to demonstrate highly dynamic epithelial-to-mesenchymal (EMT) transformation and spontaneous differentiation versus de-differentiation in 3D organotypic cultures. This phenotypic transformation is concomitant with dramatic re-arrangement of the actin cytoskeleton, regulating cell shape, plasticity and motility. These spontaneous and inducible transformations were utilised here as an experimental system to demonstrate the functionalities of the AMIDA image analysis programme.

### Screening concept, read-out and image pre-processing

A typical compound screen based on our 3D platform is outlined in Figure 1B. Cells are embedded between two layers of growth factor-reduced laminin-rich extracellular matrix (lrECM). Before compound treatments start, cells are typically cultured for 4–8 days in 3D, largely depending on the growth rate of the cell line, to promote the initiation of spheroid formation. Compound treatments are then initiated only when spheroids have reached a size suitable for continuous real-time imaging (>20 µm). The cultures are then routinely monitored for at least 6 additional days, with 1 image taken per hour. A live-cell imager like IncuCyte is optimally suited for continuous monitoring, based on phase-contrast images. However, spinning disk confocal microscopy represents the most widely used read-out for end-point analyses. Suitable endpoints for analyses have to be decided on an individual basis, and vary for different cell lines. This depends mainly on growth rates, with non-transformed cells/cell lines often growing significantly more slowly. This may require a longer duration of the experiment and/or a delayed starting point for treatments. In addition, for cells/cell lines that undergo prominent morphometric transformation, endpoints are typically selected before the end of the process ( =  complete transformation into stellate objects). For most applications, living cells are double-stained with two or more fluorescent dyes. Ideally, one dye stains metabolically active cells (e.g. Calcein-AM), the second dye dead (necrotic, membrane-damaged, e.g. ethidium homodimer) or apoptotic cells (e.g. NucView). A third dye may be included to counterstain for DNA/nuclei (SYTO62, DAPI, Hoechst).

Our read-out represents a compromise between detailed imaging of cellular/morphologic details, and fast throughput. An acceptable balance can be achieved by optimizing the image analysis program for intermediate to low magnification (5x objective) images. To focus on multicellular structures, it is only required to capture larger-scale morphological features with acceptable resolution, neglecting single-cell level details. Furthermore, imaging with low magnification expands the focal plane and fewer layers in the Z-axis are required to be imaged in order to cover the entire area of 3D cell culture. With these settings, an entire well can be imaged with a minimal number of stack images. This increases the number of individual multicellular structures captured, but conveniently reduces the number of adjacent fields required to capture the entire well. Multiple virtual sections through each multicellular structure are possible, without losing informative phenotypic details. This is sufficient to recover irregular symmetry of individual structures, or capture stellate (invasive) morphologies. The Zeiss confocal microscope used in our studies can scan 15 wells within a single ibidi μ-slide, or 96 wells of an ibidi Angiogenesis μ-plate by acquiring four image stacks of images per well (a total of 60 images/μ-slide and 240 images/μ-plate). The X and Y dimensions for a single field are approximately 4.4mm×3.3 mm, with Z ranging between 300–800 µm (at 20–40 µm intervals). The image resolution is on purpose kept at a relatively low range (672×512 pixels) to further promote fast image acquisition. The typical scanning time to scan all wells on an ibidi 15-well μ-slide is 10 minutes; a 96-well μ-plate can be imaged in 30–45 min. Prior to morphometric image analysis, nonspecific background noise is reduced by normalisation (e.g. excluding the 5 and 95 percentiles of the image distribution), followed by further background reduction, if necessary. These pre-processing procedures are performed with commercial or open source programs such as SlideBook (Intelligent Imaging Innovations) or ImageJ (NIH). As many of the subsequent operations rely on minimal background to noise ratio in the images, additional features for noise reduction were added. Background noise is caused by many factors, and can be observed as the variation of either brightness or colour inside a given structure or segment in the image. AMIDA offers several optional pre-processing features to balance, enhance or remove noise from images prior to segmentation. This variation can be countered by using Gaussian, edge enhancements, or median filtering are implemented in AMIDA.

### AMIDA basic operations

**Figure 2:**
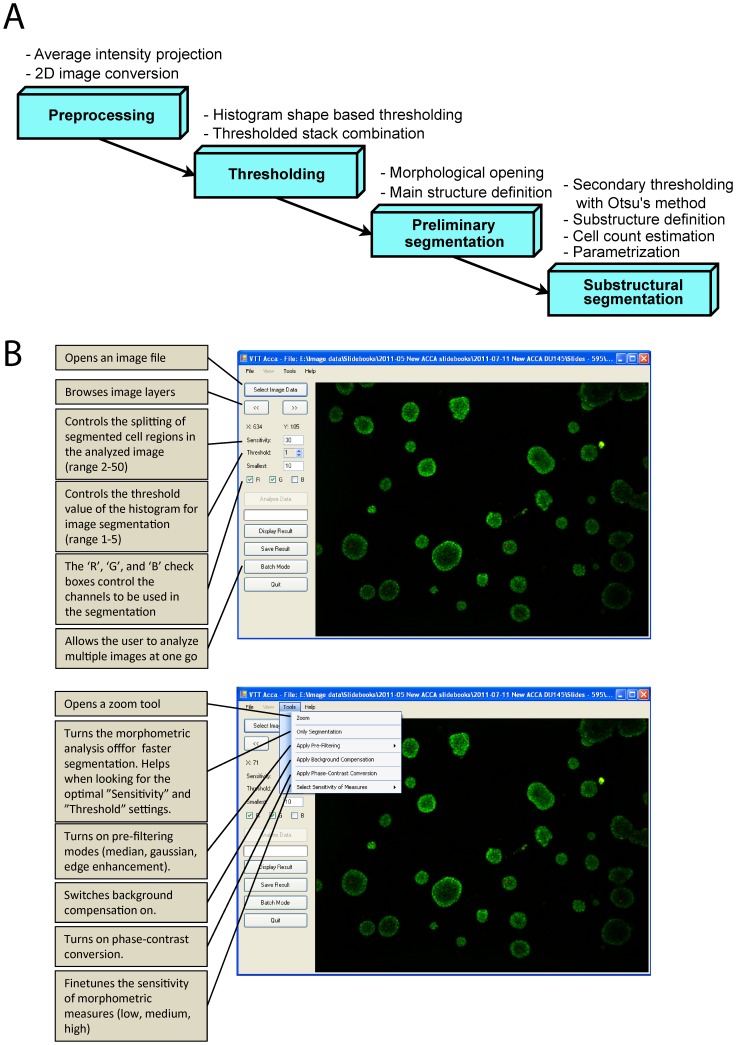
Flow diagram illustrating key functionalities of the AMIDA image analysis program. (A) Flowchart presenting four main steps in image analysis. After parameterisation all numerical data is written into an Excel file. (B) An overview of AMIDA's simple user-friendly interface and its basic operations.

AMIDA is a light multi-parametric image analysis program designed for high-content analysis of complex and heterogeneous 3D spheroid cultures (overview of functions summarized in Figure 2B and Tables 1 and 2). The program itself can be downloaded freely (AMIDA Program S1; in ZIP container format). In addition, a collection of exemplary images is available for testing its functionality can be downloaded as Image Data S1; also as a ZIP container. The AMIDA program first identifies individual multicellular structures by image segmentation, and assigns numerical values for selected cancer-relevant parameters to the objects; these are then exported as an Excel file. AMIDA was primarily designed to retrieve information from 3D confocal image stacks. However, due to the special meniscus-free 3D cell culture design, there is little spatial overlap of multicellular structures in the Z-axis, and we decided to restrict the quantitative analysis to 2D “maximum” projections of 3D images. In practice, AMIDA automatically applies an intensity projection algorithm in order to generate simple 2D raster graphics. As this may increase the overall time required for image analysis, the user can also convert 3D images into intensity projections using any other image processing program of choice (such as ImageJ, Fiji, CellProfiler, BioImageXD). The program's complete workflow can be divided into four distinct phases, as illustrated in figure 2A.

**Table 1:** List of mathematical preliminaries utilized in AMIDA.

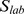	Labeled set of pixels labeled as 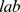 where 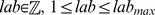 . 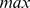 is the amount of found segmented structures where pixel 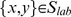 and 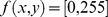 .
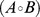	Morphological opening operation where A is a binary image matrix and B the kernel used to convolve the image. Opening can also be written as: 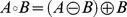 where 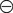 denotes erosion operation and 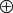 the dilation.
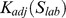	Is the adjusted kernel depending on the input set size.
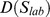	Is the set of distances from 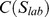 to all 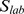 edge pixels.
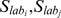	The sets of coordinates 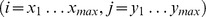 of a given labeled set of pixels.
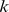	Maximum label of segmented substructures 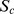 for given structure.
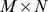	Total image size with width 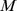 and height 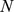 .

**Table 2:** List of morphological parameters implemented in AMIDA.

Parameter	Explanation	Formula
Area	Area of the segmented structure (in pixels)	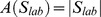
Roundness	Roundness of the segmented structure (in percentages)	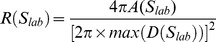
FiltRound	Filtered roundness of the segmented structure (in percentages)	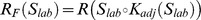
RoundDiff	Difference of the Roundness and Filtered Roundness (in percentages).	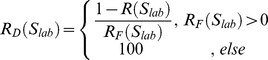
AppIndex	Index for severity of appendages of the segmented structure (no unit)	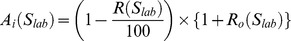
MaxApp	Estimate for the maximum length of appendages of the segmented structure (in pixels)	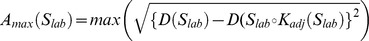
MedApp	Estimate for the median length of appendages of the segmented structure (in pixels)	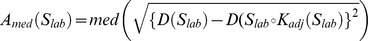
Roughness	Roughness of the surface of the segmented structure (in percentages)	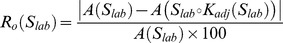
Density	Density of the segmented structure for the given colour channel (gray levels per pixel)	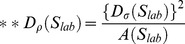
AppNumber	Estimate for the number of appendages in the segmented structure (in pieces)	----
Deviation	Standard deviation of the segmented structure (no unit)	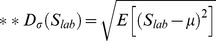
Closest	The distance of the closest neighbour of the segmented structure from the center point to the center point (in pixels)	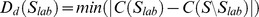
Neighbors	The number of touching neighbours of the segmented structure (in pieces)	----
SharedBound	The length of the shared boundary of all Neighbors of the structure (in pixels)	----
AreaRatio	Ratio of the structures inside the segmented structure (in percentages)	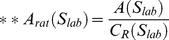
Hollowness	Estimate of the hollowness of the segmented structure for the given colour channel (in percentages)	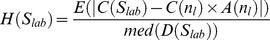
CellNumber	Estimate of the number of cells inside the segmented structure (in pieces)	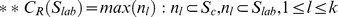
AveArea	Average area of the cells inside the segmented structure (in pixels)	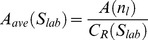
CellRatio	The ratio of the areas of the segmented structures divided by the 2D size of the imge (in percentages)	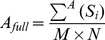
*Center of mass	The center of mass for a given segmented object (coordinate pair)	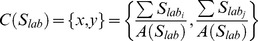
*Radius	The radius for a given structure (m depends on used sensitivity, default = 0.25)	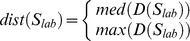

*not included in the result file.

**Computed for both channels {R,G} separately.

After pre-processing, the input image is first projected as 2D images with AIP (Average Intensity Projection) 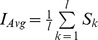 where 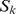 is the 3D image stack with channel stack size of 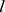. This is applied to each channel 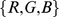 separately, resulting in a colour image 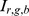. This image is then converted to grayscale by using weighed intensities from each individual channel 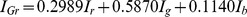. Initial image thresholding (statistiucal values reported in table S2A) applies a technique similar to the Tsai method [49], in which the valley between peaking areas is searched by a gaussian smoothed histogram function. This thresholding technique relies on the notion that the shape of the histogram remains similar throughout all of the analysed images. This phase results in a binary representation 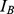 of the original grayscale image 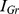 where 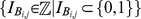, in whichpixels marked as ones (1) are considered as foreground (e.g. the cell structures) and zeros (0) as background objects. Low intensity areas inside foreground objects thus form gaps that are marked as background by thresholding. Gaps <1000 pixels are automatically filled in to construct uniform foreground segments.

In the preliminary segmentation phase, singular morphological opening 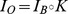 is first applied to 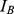
(kernel 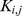 size 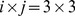) to separate structures, followed by an Eucledian distance transformation [50]. The Watershed transformation [50], [51] is then applied to the image, in order to label the main structures 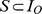
and 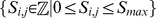. 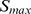 a positive integer, denoting the maximum amount of structures found. Each connected set of pixels found is labeled with an unique integer. In addition to finding the main stuctures, AMIDA uses 3D grayscale data extracted from the image stacks for each separate channel to define the actual focus plane for all individual structures 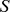. In this case, the focus information is used to further adjust the image thresholding value.

In the beginning of the substructural phase, ccell counts are computed for each structure identified, by applying the watershed transform for each 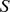 in 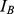. The Otsu thresholding method is used beforehand, to extract individual sets of cells 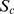, where 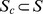. Adaptive morphological opening 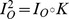 is then applied for 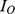 where the opening kernel size depends on the size of the corresponding structure in 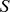. Distance transformation and watershedding are applied to 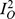 to extract structures with possible invasive appendages, and the outline variance is removed. The resulting set of structures is used to calculate several parameters describing e.g. the roughness and general variability of the structure surfaces in 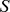. Other morphological parameters are then calculated for each structure in 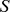. Furthermore, 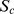
can be used to evaluate individual cell counts and localization inside the main structure and therefore to quantify the relation of apoptotic cells to non-apoptotic cells (“AreaRatioR”), or to assess the **hollownes**s and **density** of structures. AMIDA applies this substructural segmentation for both green and red channel separately. This futher contributes to the separation of apoptotic cells from live cells, which can be evaluated individually. The full program pseudo-codes for AMIDA are described in table S3. As most calculations performed for parameterisation are either constant or of linear time, the actual highest order of complexity is defined from the sub-structural segmentation phase. The watershed algorithm used is a linear time transformation procedure, applied subsequently in two distinct phases: twice for the actual structure definition in the preliminary segmentation, and again for every found 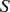 in the substructural segmentation phase with Otsu thresholding. This is raising the total highest complexity class to the quadratic scale in the worst case.

AMIDA is extremely straightforward to use. To assure proper segmentation quality, the user needs to adjust only two basic parameters: sensitivity and threshold. The sensitivity parameter controls the splitting of segmented cell regions in the analysed image. A smaller value leads to smaller segmented regions, and vice versa. The *sensitivity parameter* refers to the distance in pixels used by the watershed algorithm. In contrast, the threshold parameter controls the cut-off value of the histogram. Choosing higher *threshold values* leads to a more stringent (or too stringent) segmentation. The impact of various parameters for sensitivity and threshold are outlined in Figure S1.

AMIDA can also analyse sets of phase contrast (PC) images, e.g. from the IncuCyte instrument. Since PC pictures differ significantly from confocal images, they must be pre-processed differently prior to segmentation, and converted to fit the customized thresholding technique described above. As many of the subsequent operations rely on minimal background to noise ratio in the images, additional features for noise reduction suitable for PC images were added. Figure S2 illustrates the analysis of a set of PC images, and shows numerical quantification of image data for the morphometric effects of three compounds on tumor spheroids described later.

### AMIDA parameters

By inspecting the morphologies of spheroids formed by a panel of 25 prostate-derived cell lines [7], we devised 19 phenotypic parameters that were considered most informative and most directly linked to cancer biology. The morphometric parameters implemented in AMIDA can be divided into three classes: 1) general, 2) morphological, and 3) functional (summarized in Tables 1 and 2). Values for general and morphological parameters are derived from the same RGB channel used for structural segmentation. **General parameters** include information related to the size (*area*) of an object, its relation to neighbours (number of *neighbours*, *shared boundaries* with neighbours, *closest* neighbours), and the amount of cellular matter in relation to the local background (*cell rati*o, *average ratio*).

**Morphological parameters** include measures for features typically associated with the phenotype (habitus) of multicellular spheroids such as symmetry (*roundness*), contour *roughness* (measuring small surface features), and measures that indicate invasive processes (*appendages*). **Functional operations** are measured separately for each RGB channel. For each of the 3 channels, functional parameters can assess signal density, the number of cells per structure (*cell number*), polarisation of cells within the spheroid (“*hollowness*”), average size of cells, and the ratio of cells relative to the size of the entire spheroid.

**Figure 3:**
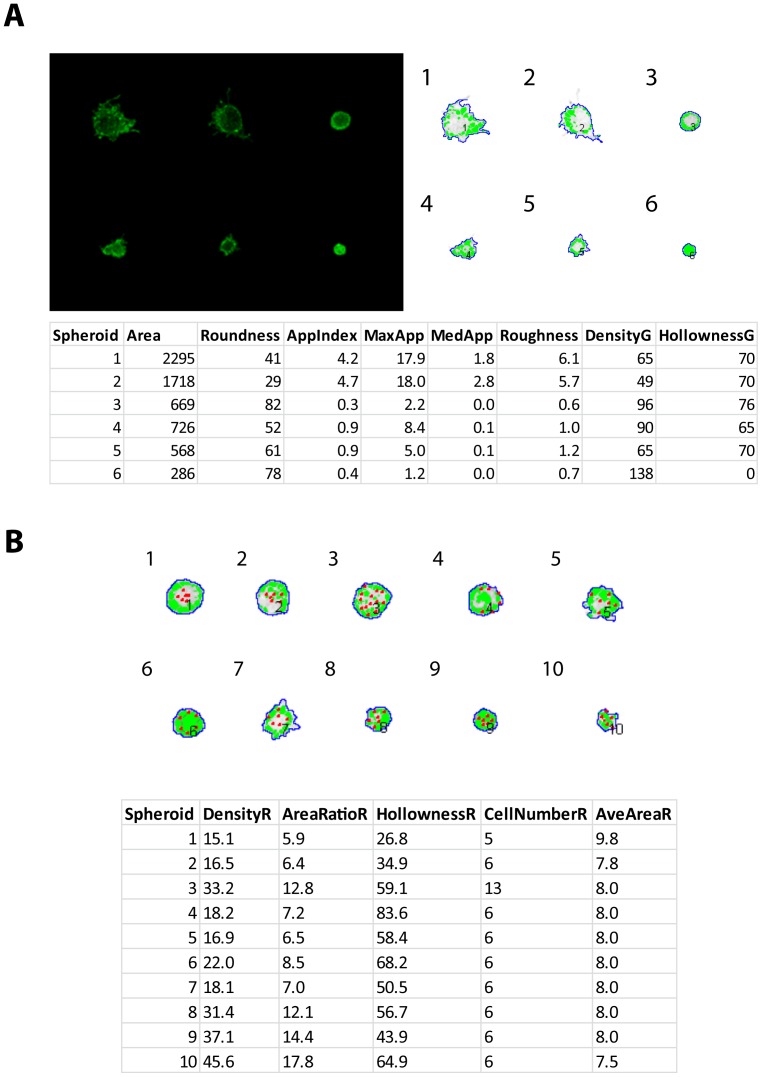
Evaluation of key parameters analysed by AMIDA. (A: left panel) Six representative PC-3 spheroids, all treated with different compounds in order to manipulate the morphology, stained for viable cells (Calcein AM) and imaged with spinning disk confocal microscope (5x objective). (A: right panel) The same spheroids segmented with AMIDA. The table in A shows numerical values appointed by AMIDA for selected morphological features. (B) Representative panel of PC-3 spheroids with red dots added by image manipulation in certain number and distribution to exemplify the power and preciseness of RGB functions (B: table).

Figure 3A (left panel) shows representative confocal microscope images of PC-3 spheroids, cultured on our miniaturised 3D Matrigel culture platform and stained with Calcein AM. The images were analysed with AMIDA (Figure 3A: right panel), and numerical data extracted for morphological and functional features. The table in Figure 3A represents the output for eight selected features. The power and accuracy of RGB channel operations is demonstrated in Figure 3B. The numerical values for five RGB channel-dependent parameters are shown on the table below.

### Data annotation and quality control

Prior to numerical data analyses, proper annotation quality controls are required. Here, data annotation refers to providing necessary experimental information, e.g. cell line names, experimental conditions, time-points, compounds and concentrations. In contrast, the main purpose of data quality control (QC) is to remove erroneously segmented structures, cell debris, staining/imaging irregularities, noise and other artefacts. QC can be performed either manually or semi-automatically, using R-based data visualisation tools. However, image quality control by visual inspection and manual intervention is extremely tedious. Our automatic QC approach is based on numerical threshold values, manually defining limits e.g. for the minimum size of objects. This implies that only objects within a certain size range will be analysed, ignoring smaller structures and debris. Nevertheless, manual visual inspection of selected images is possible and advisable. For this purpose, another specialized R script automatically discards erroneous data points according to a manually generated “excluded features” list.

### Bioinformatic tools for statistical analysis

Using our 3D cell culture platform, combined with automated microscopy and image analysis methods as described, the resulting number of individual multicellular structures captured per μ-slide can range between 1 000 and up to 5 000. This strongly depends on the cell lines utilized. Even a small scale compound study with a small number of compounds (5), experimental replicates (3) and different compound concentrations (4–5) and controls typically comprises 8–16 Ibidi μ-slides, or 2–4 96-well plates; and up to 30 different morphometric measurements are made from each spheroid imaged. Thus, the number of individual data points can easily reach into hundreds of thousands. The interpretation of complex biological responses on this scale requires robust tools for the statistical analysis and subsequent data visualization. The statistical toolset, implemented into our in-house REX interface, includes multiple scripts for heatmap and boxplot generation typically required for endpoint analyses. Additional heatmap and line graph scripts can be generated for dynamic time-lapse experiments. Heatmap visualization proved to be a particularly effective way for visualizing and comparing longitudinal drug effects and experimental conditions that result in similar morphological responses.

### Case example

**Figure 4:**
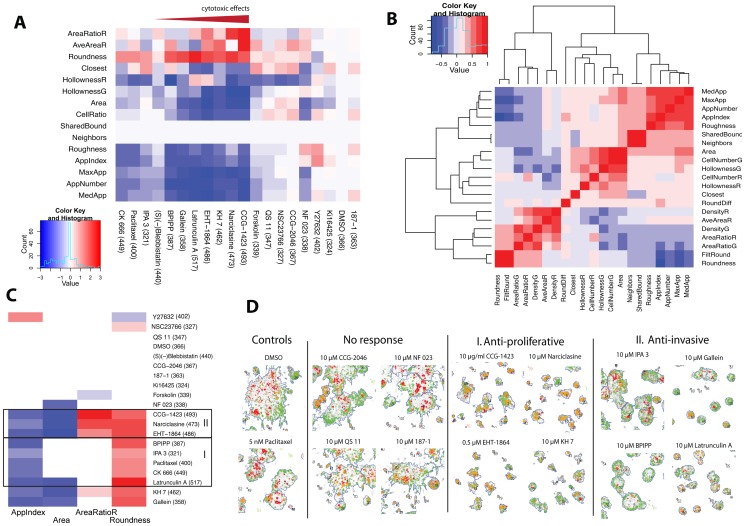
Exemplary screen based on the PC-3 spontaneous invasive transformation model. PC-3 spheroids were treated with 19 compounds mainly targeting integrity, function and organization of the actin cytoskeleton. 172–424 multicellular structures for each treatment were analysed with AMIDA program. (A) A morphometric heatmap showing standardized differences in medians between the treatments and the control for 15 morphological parameters and all 19 compound treatments. Morphological responses clustered into three functional groups. Increasing cytotoxicity, measured by the AreaRatioR parameter – based on presence of dead cells stained with ethidium homodimer - is indicated by the red gradient arrow. (B) Correlation map (nonparametric Spearman) indicating the similarity (positive correlation, red) or dissimilarity (negative correlation, blue) for 21 of AMIDAs morphometric parameters. (C) Bonferroni-corrected and Mann-Whitney U-test filtered morphometric heatmap (threshold p>0.05) focusing on four selected, most informative parameters (AppIndex, AreaRatioR, Roundness, Area). The graph highlights compounds causing mainly growth-inhibition and cytotoxicity (group II), and those that enhance spheroid symmetry and reduce number of invasive protrusions (group I). (D) The image panel shows representative, segmented PC-3 spheroids for groups I and II, compared to DMSO and paclitaxel controls, after six days of drug treatment.

For this manuscript, we performed a focused compound screen with a panel of compounds targeting the turnover and stability of the actin cytoskeleton (summarized in Table 3). As a biologically dynamic and relevant model, we used the spontaneous invasive conversion of PC-3 spheroids, which results in altered cell motility and invasiveness, and fundamental changes of cellular and multicellular morphologies. This panel of compounds includes small molecules interfering with adenylate cyclase (AC) and intracellular cyclic AMP levels, the activity of small GTPases Rac1–3, RhoA, the Rho Kinases (ROCK), PAK1 (p21 protein Cdc42/Rac-activated kinase 1), actin-regulatory proteins N-WASP, Arp2/3, myosin II, G protein signalling (via lysophosphatidic acid or LPA receptors), and the G-protein interacting protein RGS4. All of these signalling molecules act as upstream regulators of actin cytoskeletal organization. In addition, a control drug affecting primarily mitosis via tubulin polymerization (paclitaxel) was included. In addition to PC-3, another 11 cell lines (8 prostate, 3 breast derived) were included to further validate the morphological effects observed in the PC-3 model. Of the 19 drugs, 10 inhibited growth and/or invasiveness at the tested concentration, to a variable degree, with cytotoxicity measured primarily by the number of dead and dying cells inside the spheroids (red gradient symbol, Figure 4A). This illustrates that specific anti-invasive and cytotoxic effects in reality represent a continuum, with many compounds combining aspects of both. The most specific invasion-inhibitory drugs with negligible cytotoxicity at the concentrations tested were IPA3, blebbistatin, BPIPP, gallein, and latrunculin A. In contrast, ETH-1864, KH7, narciclasine and CCG-1425 showed increasing cytotoxicity (in this order). Surprisingly, paclitaxel showed remarkably low cytotoxicity at 5 nM, and clustered together with anti-invasive compounds. Next, we compared the correlation (similarity) or anti-correlation of the 26 morphological parameters used with each other for this data set (Figure 4B), in order to select the most informative ones for further streamlining the image analysis. This resulted in four, most informative key indicators (*AppIndex* and *Roundness* for invasion, *AreaRatioR* for cytotoxicity, cell death and apoptosis, and *Area* for spheroid growth), which were used to further highlight the three main response groups (Figure 4C). In group I, the most specific invasion inhibitory drug effects were clustered together, showing negligible cytotoxicity. Group II, in contrast, contained the 3 compounds with the highest cytotoxicity. Group III (unmarked) contains the rest of the compounds without prominent effects on invasion or growth. These four key indicators may therefore be sufficient to distinguish inactive compounds from active drugs that impact on proliferation, cytotoxicity, and modulators of tumor cell invasion. These effects are further documented in (Figure 4D). In group II, the RhoA-modulators CCG-1423, narciclasine, the pan-Rac inhibitor EHT-1864 as well as the adenylate-cyclase inhibitor KH7 mainly resulted in cytotoxic effects, which in turn are likely to impair tumor cell invasion. In contrast, drugs in group I including the general G-protein inhibitor gallein, which affects Gβγ-dependent cellular activities, the specific PAK1 inhibitor IPA3, the non-competitive guanylyl cyclase (GC) and adenylyl cyclase (AC) inhibitor BPIPP, and latrunculin A, a reversible inhibitor of actin assembly which also blocks actin adenine nucleotide exchange, primarily inhibited invasion with negligible effects on growth.

**Table 3:** List of compounds used in exemplary screens.

Pathway	Drug	Mechanism of action	Concentration
**cAMP pathway**	KH 7	Adenylate cyclase inhibitor	10 µM
	BPIPP	Adenylate cyclase inhibitor	10 µM
	Forskolin	cAMP activator	10 µM
**RhoA pathway**	CCG-1423	RhoA inhibitor	10 µg/ml
	Narciclasine	RhoA activator	10 µM
	Y-27632	ROCK1/2 inhibitor	10 µM
**Rac pathway**	NSC23766	Rac1 inhibitor	10 µM
	EHT-1864	Rac 1, Rac2, Rac3 inhibitor	0.5 µM
	IPA 3	class I PAK (PAK1–3) inhibitor	10 µM
**G-protein signalling**	Gallein	G protein βγ inhibitor	10 µM
	NF023	G protein α o/i inhibitor	10 µM
	QS 11	GTPase activating protein of ADP-ribosylation factor 1 inhibitor	10 µM
	CCG-2046	RGS4 inhibitor	10 µM
**Actin/myosin**	(S)-(-)Blebbistatin	Myosin II inhibitor	10 µM
	CK 666	Arp2/3 complex inhibitor	10 µM
	187-1	N-WASP inhibitor	10 µM
	Latrunculin A	Actin adenine nucleotide exchange inhibitor, actin monomer sequestering agent	10 µM
**LPAR1/3**	Ki16425	LPAR1/3 antagonist	10 µM
**Mitosis**	Paclitaxel	Spindle-assembly inhibitor	5 nM

### Quality control and reproducibility

**Figure 5:**
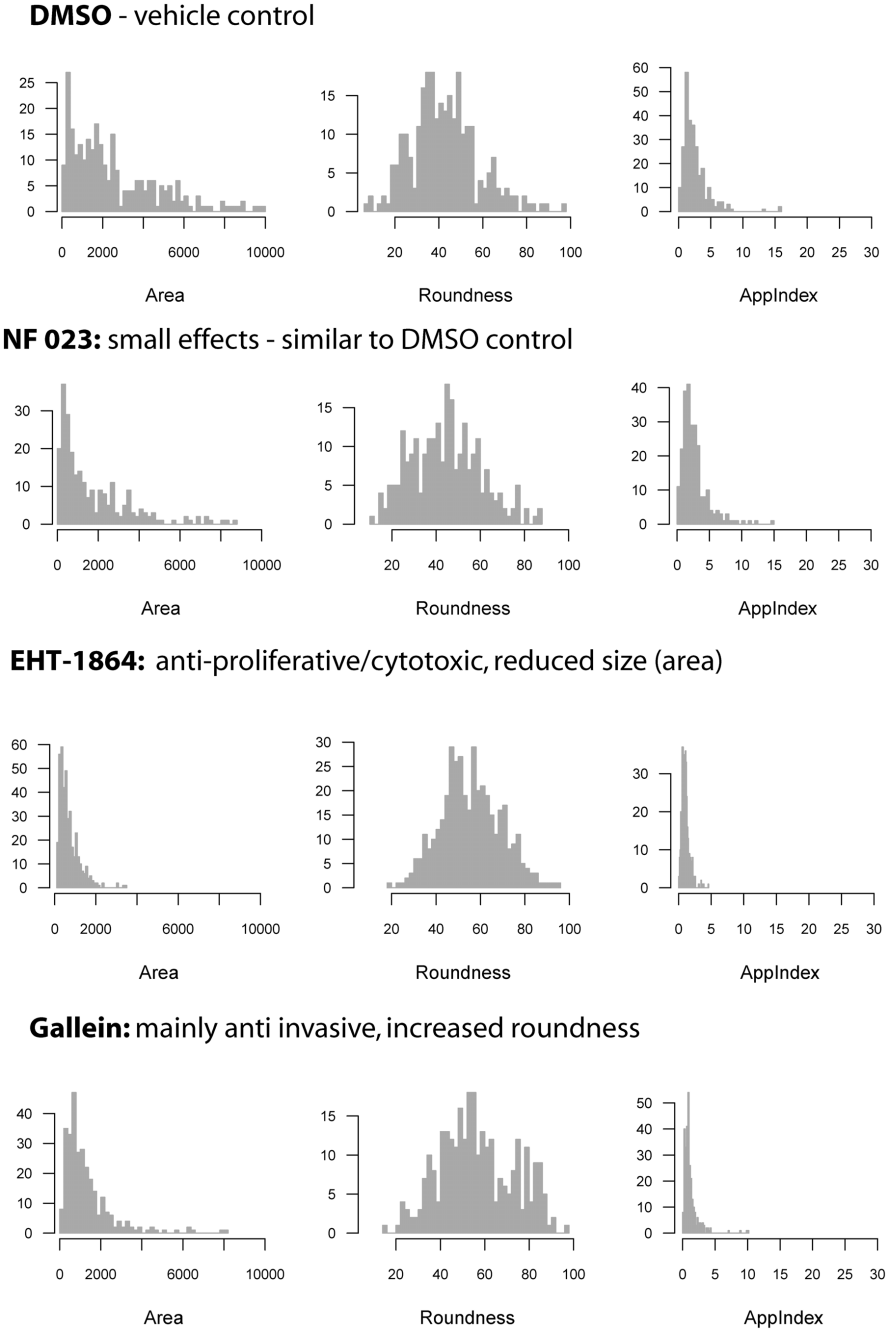
Histograms showing the distribution of morphological response data in the exemplary screen. The data is shown for three key parameters, Area (representing spheroid size), Roundness and AppIndex (representing symmetry), and for three experimental compounds each one representing one of the response groups (DMSO: control, NF023: no response, EHT-1864: growth inhibition, gallein: anti-invasive).

One strategy to monitor assay quality is based on examining the distribution of data, in order to detect outliers which indicate potential artefacts and incorrectly segmented images. These may appear as off-centre peaks or tails in histograms, and can be easily identified and discarded. Histograms are particularly useful to visualise the extent, heterogeneity and kinetics of drug responses. Figure 5 illustrates characteristic data distribution for three key parameters (*Area*, *Roundness* and *AppIndex*) and three example compounds, each representative for one of the response groups. Similar to the DMSO controls, NF-023 treatment had no detectable effect on invasive PC-3 spheroids. In contrast, the Rac-inhibitor EHT-1864 shows combined growth and invasion-blocking effects, indicated by shifting the peaks of the *Area* and *AppIndex* histograms to the left, together with a shift of the *Roundness* peak to the right. The relatively invasion-specific G-protein inhibitor gallein shifts the peak(s) of the *Roundness* histogram even further to the right. In addition, the possible emergence of a population of particularly well-rounded spheroids (with a peak around 80% *Roundness*) is indicated. Spheroid size (*Area*) is only marginally affected by gallein.

### Statistical evaluation of robustness and reproducibility

All compound treatments were done in triplicate, with four fields or image positions imaged per well. We assessed the reproducibility (robustness) of our analysis by measuring the impact on variance, by switching between *Wells* and *Positions*. This was accomplished by fitting a linear mixed model on the data, using these variables as random effects. The model used was of the form:
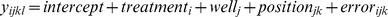
where 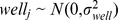, 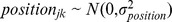, 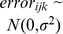. The estimates for 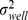 and 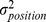 can be compared to the variance of the unexplained random noise 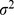, in order to evaluate consistency between experiments. Table S2 shows the estimates for three different morphological parameters, Size (measured as logarithmic *Area*), *Roundness*, and logarithmic *AppIndex*, for a total of 10 cell lines treated with the entire set of drugs. The variance between *Wells* and *Positions* is consistently smaller than the residual error, although not completely negligible. Based on these results, we conclude that our 3D platform, used in conjunction with automated image analysis, is sufficiently robust and reproducible, with marginal well-to-well variation and noise. Nevertheless, performing relevant replicates remains a critical issue. We are confident that conclusions concerning altered morphology as the result of biological and/or chemical perturbations can be exclusively based on true observations, only marginally affected by the intrinsic variation between replicates.

### Experimental validation

**Figure 6:**
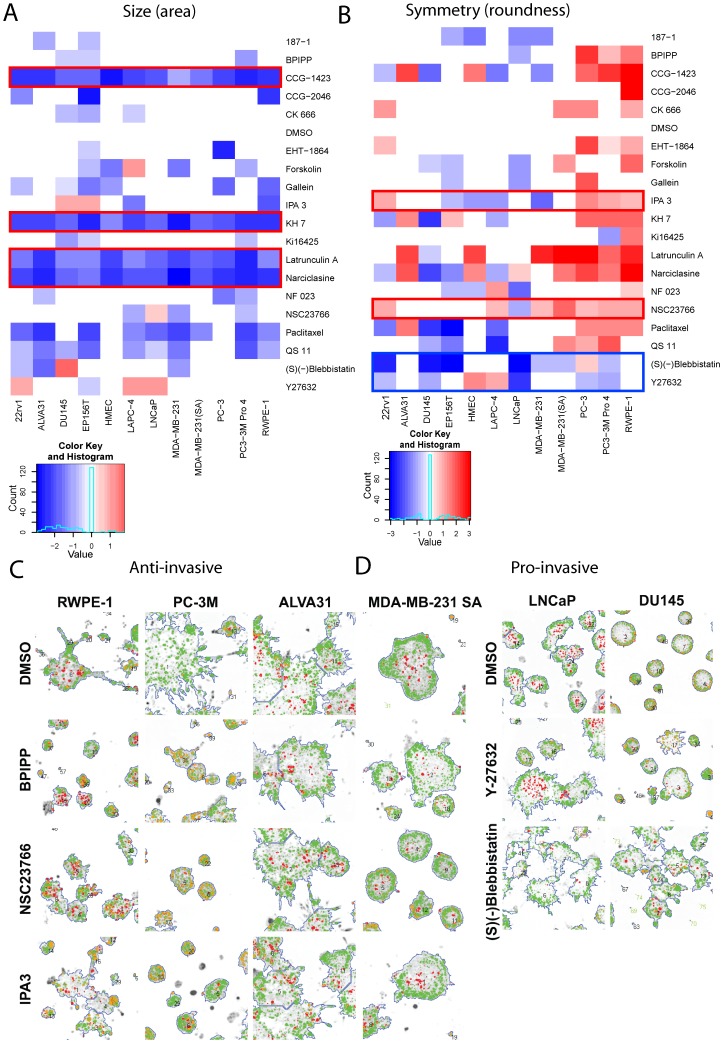
Validation of morphological responses with 9 additional prostate and 3 breast cancer cell lines. The heatmaps illustrate changes in spheroid growth (A: Area) and general symmetry (B: Roundness) in response to the 19 compound treatments (5031–16415 multicellular structures analysed for each cell line). (A) CCG-1423, KH7, latrunculin A and narciclasine are preferentially cytotoxic and/or antiproliferative compounds across all cell lines, as highlighted by red boxes. Paclitaxel, at a concentration of 5 nM, shows partial cytotoxic/antiproliferative effects only in some of the cell lines. (B) Effects of mainly anti-invasive compounds IPA3 or NSC23766 were reproducible in many of the spontaneously invasive (or branching) cell lines PC-3, PC-3M Pro4, and RWPE-1. (C) Images segmented and analysed with AMIDA. Effective anti-invasive functions of the compounds IPA3, BPIPP and NSC23766 against the most aggressive, motile and invasive cell lines PC-3M, ALVA31, MDA-MB-231 (both SA and parental ATCC), and RWPE1. The extremely invasive PC3-derivative ALVA31 was not affected, however. (D) Blebbistatin and Y-27632 show invasion-inducing function in spheroids formed by the LNCaP and DU145 lines which typically form round spheroids and lack invasive properties.

We set out to further validate the drug response patterns observed in PC-3 cells, by repeating the compound treatments with 11 additional prostate and breast derived cell lines from malignant and benign/non-transformed origin. With the exception of the pan-Rac-inhibitor EHT-1864, all drugs that showed anti-proliferative effects in PC-3 (CCG-1423, KH-7, latrunculin A and narciclasine; highlighted by red boxes) also inhibited spheroid growth across the entire panel of cell lines (Figure 6A). The 3 most effective anti-proliferative compounds in 3D were also most effective in 2D conditions (72 h), acting in a dose-dependent fashion (EHT-1864, KH7, narciclasine; to a lesser degree CK-666; Figure S4A). To validate induction of programmed cell death in 3D, we also measured apoptosis after 72 h of drug treatment in 2D (Figure S4B and S4C). The 2D results were largely in agreement with 3D measurements: 10 µM Narciclasine promoted apoptosis by almost 500% (p = 3.1×10−5), while 10 µM KH7 only resulted in a non-significant increase of only 20% (p = 0.3), and BPIPP had marginal effects (Figure S4C). These growth-inhibitory and pro-apoptotic effects may be primarily due to cytotoxicity and increasingly relevant at higher concentrations (>1 to 5 µM).

The anti-invasive and differentiation-promoting effects, shown here by increased spheroid symmetry or roundness, exerted by the non-competitive guanylyl cyclase (GC) and adenylyl cyclase (AC) inhibitors BPIPP and KH7, and PAK1-inhibitor IPA3 are clearly seen on invasive (PC3, PC-3M Pro 4), and branching RWPE-1 cells, also to some degree on the breast cancer line MDA-MB-231 (Figure 6B). This is further exemplified in Figure 6C, showing the strong invasion-blocking results of BPIPP, NSC23766 and IPA3 on the RWPE1 line that forms branching structures, and the successively more invasive, PC3 derivative lines PC3M as well as a metastatic variant of the MDA-MB-231 breast cancer line. In PC-3, PC3M, RWPE1 and MDA-MB-231 cells, NSC23766 was as effective and specific as IPA3. NSC23766 is a selective inhibitor of Rac1-GEF interaction and prevents Rac1 activation by Rac-specific guanine nucleotide exchange factors (GEFs). It inhibits Rac1-mediated cell functions and was reported to reverse tumor cell invasiveness in prostate cancer cells. In accordance with the assumed invasion-specific effects, the compounds BPIPP, IPA3 and gallein did not have a strong effect on proliferation in 2D (Figure S4A).

Dose-dependent and possibly specific, anti-invasive versus cytotoxic effects of Rac and Rac-related inhibitors on PC3 spheroids were further examined, as shown in Figure S5. NSC23766, EHT-1864 and IPA3 all directly or indirectly inhibit Rac family GTPases and have strong effects on PC-3 spheroid growth and invasion. We proceeded to further test and compare these to two additional selective Rac1 inhibitors (Rac inhibitor I, Merck #553502; Rac inhibitor II, Merck #553511) and one indirect Rac1 inhibitor (ITX3), using three concentrations. IPA3 and EHT-1864 showed linear dose-response in PC3 cells, resulting in increased roundness coupled with incremental loss of appendages ( =  invasive structures) but also a significant reduction of spheroid size and therefore cytotoxic/growth inhibitory effects. EHT-1864 was clearly toxic already at micro-molar concentration. NSC23766, ITX3 and Rac inhibitor II blocked invasion only in the higher micro-molar concentrations.

Furthermore, the pro-invasive effects of the ROCK inhibitor Y-27632 and the myosin-II-inhibitor (-)-(S)-blebbistatin specifically promoted invasion in otherwise non-invasive, transformed cancer lines. These preferentially form round spheroids surrounded by partially or completely intact basement membranes (22rV1, LNCaP, DU145). This indicates that ROCK inhibitors have a profound effect on multicellular integrity and perturb epithelial maturation (Figure 6D) by interfering with the formation and organization of the actomyosin cytoskeleton, specifically in tumor cells. Similarly, blebbistatin is a selective inhibitor of myosin II ATPase and blocks myosin in an actin-detached state. Both compounds prevent functional actomyosin cross-linking.

### Cell motility in 2D versus 3D invasion

In order to assess cell motility and invasion through lrECM in 2D monolayer culture, we applied two complementary migration assays. We compared standard scratch-wound migration assays on uncoated plastic plates (Figure S6A), with a modified invasion assay using plates coated with GFR-Matrigel (Figure S6B). Here, the 2D monolayer of cells grown on a homogeneous layer of ECM is first “wounded”, and subsequently covered with a second layer of lrECM. In contrast to plastic plates, cells need to actively invade through a mesh of functional lrECM to close the wound. Two of the most significant treatments were selected: the AC inhibitor BPIPP and the Rac-downstream PAK1 inhibitor IPA3 (Figure S6A and S6B); both effectively blocking 3D invasion without cytotoxicity. The results were strikingly inconsistent: BPIPP had no measurable effect on 2D cell migration on plastic (Figure S6C top), but markedly delayed wound closure in the Matrigel-coated invasion assay (Figure S6D top), which is more closely related to the 3D culture settings. In contrast, IPA3 was extremely effective in both assays and blocked invasion in both 2D and 3D, (Figure S6C and S6D).

Table 4 further summarizes the outcome of 2D monolayer and 3D organotypic assays. In most cases, 2D proliferation and 3D spheroid growth (*Area* or *CellRatio* readout) matched reasonably well, as did 3D *AreaRatioR* and apoptosis measured in 2D. However, the results from three different invasion assays were controversial, possibly indicating different modes of cell motility employed in various ECM and microenvironment. Two compounds showed no measurable effects (NSC23766, BPIPP) in standard 2D motility assays, while IPA3 decreased cell motility. In contrast, all three compounds significantly decreased motility in 2D invasion assays through Matrigel, in good agreement with the findings from the 3D assays.

**Table 4:** Summary of drug treatments of PC-3 cells in monolayer and organotypic culture.

Proliferation	EHT-1864 (0.5 µM)	KH7	CCG-1423 (10 µg/ml)	Narciclasine
2D proliferation	99%	70%	55%	23%
	p = 0.000	p = 0.016	p = 0.010	p = 0.000
2D apoptosis	ND	120%	ND	483%
		p = 0.325		p = 0.000
3D size (Area)	38%	52%	48%	48%
	p = 0.000	p = 0.000	p = 0.000	p = 0.000
3D cell ratio (CellRatio)	33%	45%	41%	45%
	p = 0.000	p = 0.000	p = 0.000	p = 0.000
3D apoptosis (AreaRatioR)	150%	118%	209%	160%
	p = 0.000	p = 0.006	p = 0.000	p = 0.000
Drug Effects in 2D versus 3D conditions				
**Invasion**	**NSC23766**	**IPA3**	**BPIPP**	
2D migration	No effect	Decreased	No effect	
2D Matrigel invasion	Decreased	Decreased	Decreased	
3D invasion (AppIndex)	Decreased	Decreased	Decreased	

## Discussion

Despite many technical advances, some of the most informative aspects of 3D phenotypes, such as their complexity and heterogeneity, remain difficult to quantitate. The lack of straightforward, automated, user-friendly and fast 3D platforms, assisted by specific 3D image analysis tools, affects the practicality of phenotypic high-content screening (HCS) assays.

To demonstrate the potential of the 3D platform combined with automated image analyses and statistics, we utilized small-molecule inhibitors to modulate pathways involved in re-organization of the actin cytoskeleton, in particular transformation of differentiated “round/acinar” into invasive “stellate” spheroids [6], [7], [52]. The library of 19 small-molecule inhibitors specifically modulated upstream mechanisms of actin cytoskeleton turnover and stability, and was further used to compare the effects of invasion in 3D with cell motility in 2D. The mesenchymal phenotype observed in spontaneously invading PC-3 cells may reflect a spontaneous EMT (epithelial-to-mesenchymal transformation), executed by re-arrangement of the actin cytoskeleton combined with promotion of adhesion-dependent processes. Mesenchymal invasion may affect single cells or chain- or string-like multicellular threads of cells (reviewed in [29], [33]); the latter being observed e.g. in PC-3 cells. blebbistatin, a myosin II inhibitor, directly interfered with the cortical actin cytoskeleton and increased invasiveness. In contrast, latrunculin A, an actin-adenine nucleotide exchange inhibitor, and CK-666, an Arp2/3 complex inhibitor, suppressed formation of invasive mesenchymal structures and enhanced *Roundness* by blocking actin polymerization. The dual adenylyl-cyclase (AC) and guanylyl-cyclase (GC) inhibitor BPIPP selectively blocked formation of invasion and mesenchymal protrusions across all invasive cell lines Rho and Rac GTPases are downstream effectors of G-protein signalling. The non-specific G-protein Gβγ inhibitor gallein, but not the specific Gαo inhibitors NF-023 nor QS-11, an inhibitor of GTPase-activating ADP-ribosylation factor 1, blocked mesenchymal processes, nor did the LPAR1-antagonist Ki-16425 which blocks signalling upstream of G-proteins Gα12/13 and Gαi. In contrast, compounds interfering with RhoA signalling, such as blocking downstream ROCK kinases (ROCK inhibitor Y-27632), specifically impeded epithelial polarization in all transformed cells/spheroids, causing enhanced motility. Even non-invasive cell lines like LNCaP and DU145 form invasive structures after ROCK inhibition. Direct interference with RhoA, e.g. by the RhoA-activator narciclasine and RhoA-inhibitor CCG-1423, caused apoptosis across almost all 12 cell lines tested. This indicates multiple roles for RhoA besides cell motility in spheroid differentiation, survival signalling and cell proliferation. Rac and Cdc42 counteract RhoA signalling pathways and promote tumour cell invasion [38], [53]. Accordingly, the selective Rac1-inhibitor NSC23766 improved round symmetry in 7 of the 12 cell lines, without marked cytotoxicity, and was also consistently efficient in 2D cell migration assays. In contrast, the pan-Rac inhibitor EHT-1864 prevented formation of invasive structures at nanomolar range, but induced apoptosis at higher concentrations. Additional evidence for the key role of Rac activation in the invasive switch and mesenchymal invasion versus actomyosin-contractility comes from Rac inhibitors I and II (Merck #553502 and #553511) of which the latter one blocked invasion effectively at micromolar range. Furthermore, the data from blocking Rac regulators attest to these findings: IPA-3 inhibits Rac signalling by blocking all three group-I p21-activated kinases (PAK1–3), and most consistently decreased cell-invasion across all 2D and 3D invasion assays. In our set of experiments, mesenchymal invasion appears mainly supported by RAC small GTPases (RAC1–3) and downstream PAKs [54], [55], while epithelial integrity and epithelial motility were promoted by RhoA and its specific downstream signalling mechanisms (ROCK kinases, myosin-II).

The 3D platform described here is based on the potential of single epithelial (tumour) cells, embedded between two layers of relevant matrix, to form a broad spectrum of polarized and differentiated spheroids – according to the individual cells' intrinsic differentiation potential. This “clonal” approach is in contrast to the re-aggregation models introduced earlier. These do not reflect the growth properties of individual tumor cells, and often only one spheroid is formed per well which does not support statistical evaluation. Thus, the main benefit of our sandwich platform is the formation and development of hundreds of independent spheroids in parallel, which can be readily imaged by confocal or phase contrast microscopes. In addition, spheroid development is limited within a single optical plane, supporting automated microscopic imaging, and reducing the number of image layers required to capture the entire growth area. Provided single cells can be successfully separated and seeded, the clonal approach effectively has the potential to recapitulate intrinsic tumor cell heterogeneity and dynamic features. The sandwich-style setup is optimally suited to monitor different modes of cell motility in real-time; and also suitable for tumour/stroma co-culture settings (not shown). The phenotypic analysis of hundreds of multicellular structures in parallel, within a single experiment or well, from hundreds of wells in parallel allows statistically significant conclusions about heterogeneity and tumor cell plasticity. Ultimately, this strategy allows us to indirectly address the genetic variability contained in cell populations, or the drift that occurs for example in long-term drug exposures or other functional experiments. The use of pre-fabricated cell culture plates for automated microscopic imaging, combined with standardized matrix deposition and cell seeding protocols favours assay miniaturization and standardization. In combination, this strategy allows massively parallel imaging and quantitative measurements e.g. of time- and dose-response courses within a large-scale experimental set-up, at reduced cost.

A useful platform for 3D assays ideally requires the combination standardized cell culture settings with suitable software solutions, which must be capable to analyse the massive amount of microscopic images generated in a single compound screen. The AMIDA software was developed to directly address these needs, specifically focusing on the analysis of those phenotypic features that are primarily relevant for multicellular 3D cultures. Our 3D platform, combined with AMIDA represents a practical compromise between sufficient but not overarching image resolution and computational effort, required for numerical quantitation of massive numbers of structures. This practical compromise allows high-content assays with reasonable throughput, considerable scale and acceptable cost. AMIDA addresses all of these aspects, and represents a user-friendly, particularly lean software solution that requires very little user-interaction and optimization of the analytical parameters. This is in striking contrast to other image analysis programs that may be overall more flexible, but considerably more difficult to fine-tune and optimize for the task of analysing 3D images. Several research groups have developed proprietary software scripts or plug-ins for existing image-analysis programs such as CellProfiler [46], [56], however usually focused on single-cell analyses. In addition, several commercial software packages have been released, mainly for the pharmaceutical HCS market, including VoxelView (SGI Vital Images; www.vitalimages.com), Imaris (www.bitplane.com), Metamorph (Molecular Devices; www.moleculardevices.com), Definiens (www.definiens.com), Analysis (Olympus, OSIS; www.soft-imaging.de), and Volocity (PerkinElmer; www.perkinelmer.com) [57], [58]. Invariably, these programs are specialized on the detailed analysis of few selected 3D spheroids or histology but not specialized on the analysis of thousands of structures. In this study, we introduce an integrated image analysis software program AMIDA (**A**utomated **M**orphometric **I**mage **D**ata **A**nalysis), which was specifically developed for this purpose. AMIDA works with both single (and projected) images as well as original stacks of confocal images of different resolution and multiple colours channels but also processes black and white phase-contrast images. AMIDA returns the most relevant phenotypic features such as spheroid size, shape and geometry, invasive features, surface and internal structures, or apoptosis as quantitative measurements. The special focus of AMIDA is on quantitation of dynamic features, such as formation of invasive cellular protrusions. This allows us to accurately measure the onset of invasion and distinguish different modes of tumor cell motility. Compared to open-source or commercial software applications, AMIDA has a limited scope (entirely specialized on 3D image analyses), but is very user friendly with few adjustable parameters. The morphometric measures introduced here may also be utilized in industry-standard plate readers used for high-content screening, such as Opera/Operetta (PerkinElmer), or InCell 6000 (GE Health Care), widely used in industry and contract-research. We have implemented novel measures for multi- and subcellular structures, metrics based on the shape or interior of the segmented structures; and additional informative measures can be easily implemented – provided they can be mathematically defined. Currently, all morphometric calculations are based on key biological events relevant for cancer research, such as differentiation, apoptosis and invasion, similar to earlier reports [59]. Our simple morphometric measures are generally intuitive, and relate to the underlying biological processes in a more natural fashion than theoretically complex metrics. Nevertheless, higher-order, descriptive mathematical metrics can be implemented, thus opening the possibility to quantitate additional structural aspects that are currently beyond human recognition.

## Supporting Information

Figure S1 **Impact of modifying the AMIDA program parameters “sensitivity” and “threshold” on segmentation.** (A) An image of PC-3 cells cultured 10 days in 3D was used as an example (A: left image). Modifying “sensitivity” from values of 5 to 40 (with constant threshold 1) results in reduced fragmentation of adjacent spheroid structures (A: upper right panel). The “threshold” parameter has opposite effect: increasing the value from 1 to 5 (with constant sensitivity at 20) has a notable effect on fragmentation (A: lower right panel). (B) The effect of modifying “sensitivity” and “threshold” parameters was statistically evaluated by Kruskal-Willis rank sum test. As expected, increasing the “sensitivity” value yields larger (p = 0) and fewer cellular structures (sensitivity 5: N = 152, sensitivity 40: N = 37). However, symmetry (Roundness) is not significantly affected. A higher threshold value tends to identify more structures (threshold 1: N = 56, threshold 5: N = 65) but has no significant effect on structure size or symmetry measures in this case.
(TIF)

Figure S2 **Impact of modifying the AMIDA “sensitivity” parameter on a whole experiment level.** Non-invasive DU145 cells/spheroids were cultured 4 days in 3D Matrigel matrix and exposed to 19 different compounds for 6 days. The 3D cell cultures were imaged with spinning disk confocal microscope and the maximum intensity projection images were analysed using three different sensitivity settings at (A) = 10, (B) = 20 and (C) = 40 (threshold: constant setting at t = 1, size >100 pixels). The heatmaps show the standardized, p-value filtered (Bonferroni-corrected Mann-Whitney U-test p<0.05) differences in medians between treatments and DMSO controls for the selected features. Both the treatments and the morphological parameters are hierarchically clustered based on complete linkage of Euclidean distances, enabling unbiased evaluation. The total number of observations ( =  spheroids) for each treatment is indicated in parentheses. “Sensitivity” values of 20 and 40 yield almost identical clusters, whereas the value 10 stands out as clearly different, most probably because of heavier fragmentation.
(TIF)

Figure S3 **Exemplary evaluation of segmentation and image analysis of phase contrast images, using AMIDA.** (A) Original phase contrast images as derived from IncuCyte (left), and after background subtraction and segmentation (right). (B) Time course of spheroid growth (left graph) for control (DMSO) compared to two compound treatments (BPIPP and IPA3) known to primarily affect tumor cell invasiveness. With DMSO, most spheroids undergo invasive transformation after 100 h of treatment, which is partly inhibited by BPIPP and IPA3 (right graph).
(TIF)

Figure S4 **Validation of dynamic responses observed in 3D culture, using standard 2D monolayer assays.** (A) Proliferation: PC-3 cells were treated for 72 h with 4 concentrations of each compound. Cell numbers were assessed by nuclear staining with Hoechst (results shown as percentage of the DMSO control, 204–1841 nuclei counted per treatment). (B) Apoptosis: PC3 cells were treated in 2D monolayer with three compounds that induce apoptosis in 3D settings, namely adenylate-cyclase inhibitors BPIPP and KH7, and RhoA activator narciclasine, and stained with NucView 488 caspase-3 substrate to detect apoptotic nuclei. (C) Apoptosis was quantified from 2D image data using IncuCyte (2011A Rev2) object counting tool (v2.0). The quantification indicates that narciclasine massively induces programmed cell death, while all other drugs only result in small increases of apoptosis at the highest (10 µM) concentrations.
(TIF)

Figure S5 **Evaluation of anti-invasive effects of several Rac-related inhibitors on PC-3 cells cultured in 3D Matrigel matrix for 10 days.** (A) Spinning disk confocal microscope (5x objective) image projections of PC-3 spheroids exposed to six inhibitors – namely IPA3 (Group I p21-activated kinase or PAK inhibitor), EHT-184 (non-selective Rac family GTPase inhibitor), NSC23766 (selective Rac1-GEF inhibitor), ITX3 (selective TrioN RhoGEF inhibitor), Rac inhibitor I (Merck #553502) and Rac inhibitor II (Merck #553511) – all in three concentrations (0.5, 1 and 10 µM) for six days (days 4-10), stained at day 10 with calcein AM live cell colour. (B) A heatmap of AMIDA generated morphometric data displaying p-value filtered (Mann-Whitney U-test, Bonferroni-corrected cut-off p<0.05) standardized median differences across 10 selected morphological features. (C) Boxplots highlighting clear dose-responses for spheroid size and invasiveness in response to several Rac-related inhibitors, most notably IPA3, EHT-1864, NSC23766, ITX3 and Rac inhibitor II.
(TIF)

Figure S6 **Validation of altered cell migration and motility measured in 2D and 3D, using PC3 cells.** (A) 2D Scratch wound migration and (B) 3D invasion assays in Matrigel, treated with the IPA3 compound. (C and D) Quantification of cell motility in 2D cultures using IncuCyte (2010A Rev2), treated with compounds that were most specifically active invasion suppressors in 3D: adenylate-cyclase inhibitor BPIPP and PAK-class I inhibitor IPA3. Compounds were administered in two different concentrations. (C) In the 2D migration assays, a confluent PC-3 monolayer cultured on Essen ImageLock plates was wounded with Essen CellPlayer, wound closure monitored for 24 h, and quantified by IncuCyte imaging. The wound closure was measured as wound cell density in relation to the original wound area. (D) In 3D invasion assays, confluent cell layers were scratched on Matrigel-coated ImageLock plates and covered by an additional layer of Matrigel, containing the compounds. Wound closure was monitored for 112 h, and quantified with IncuCyte. Time series illustrating delayed wound closure in response to IPA3, a PAK1 inhibitor, both in 2D migration and invasion assays.
(TIF)

Table S1 **List of all cell lines used in the validation screens.**
(DOCX)

Table S2 **Estimated standard deviation parameter values for random effects.** The values are shown for three morphological parameters, logarithmic Area, Roundness and logarithmic.
(DOCX)

Table S3 **A summary of pseudo codes used in AMIDA.**
(DOCX)

AMIDA Program S1 **Compressed ZIP file that contains the AMIDA program (as. exe file format) for computers with both 16-bit (Subfolder x86) and 8-bit based microprocessors (subfolder x64).** In addition, a supplemental. dll file (is included in both subfolders. This file may be required by some computers to run AMIDA properly. AMIDA is started by double clicking the amida.exe file. The correct folder corresponding to the users' version of windows has to be chosen. (Newer computers have a 64-bit (x64) instruction set while older often still have a 32-bit (x86) set. A single image file (e.g. own data, or exemplary 3D images from Supplemental Image Data file S5) can be chosen for analysis by clicking the ‘Select Image Data’ button from the AMIDA user interface. Clicking the ‘Analyze Data’ button start the analysis.
(ZIP)

Image Data S1 **Compressed ZIP file contains a set of exemplary test images derived from 3D cultures of HeLa and PC3 cells, in different formats and resolutions.** These images can be analysed with the AMIDA software.
(ZIP)
