# Supplementary figures and images for: Quantification of Dynamic Morphological Drug Responses in 3D Organotypic Cell Cultures by Automated Image Analysis
Source: PLoS One. 2014 May 8;9(5):e96426. doi: 10.1371/journal.pone.0096426 (PMC4014501; doi:10.1371/journal.pone.0096426)

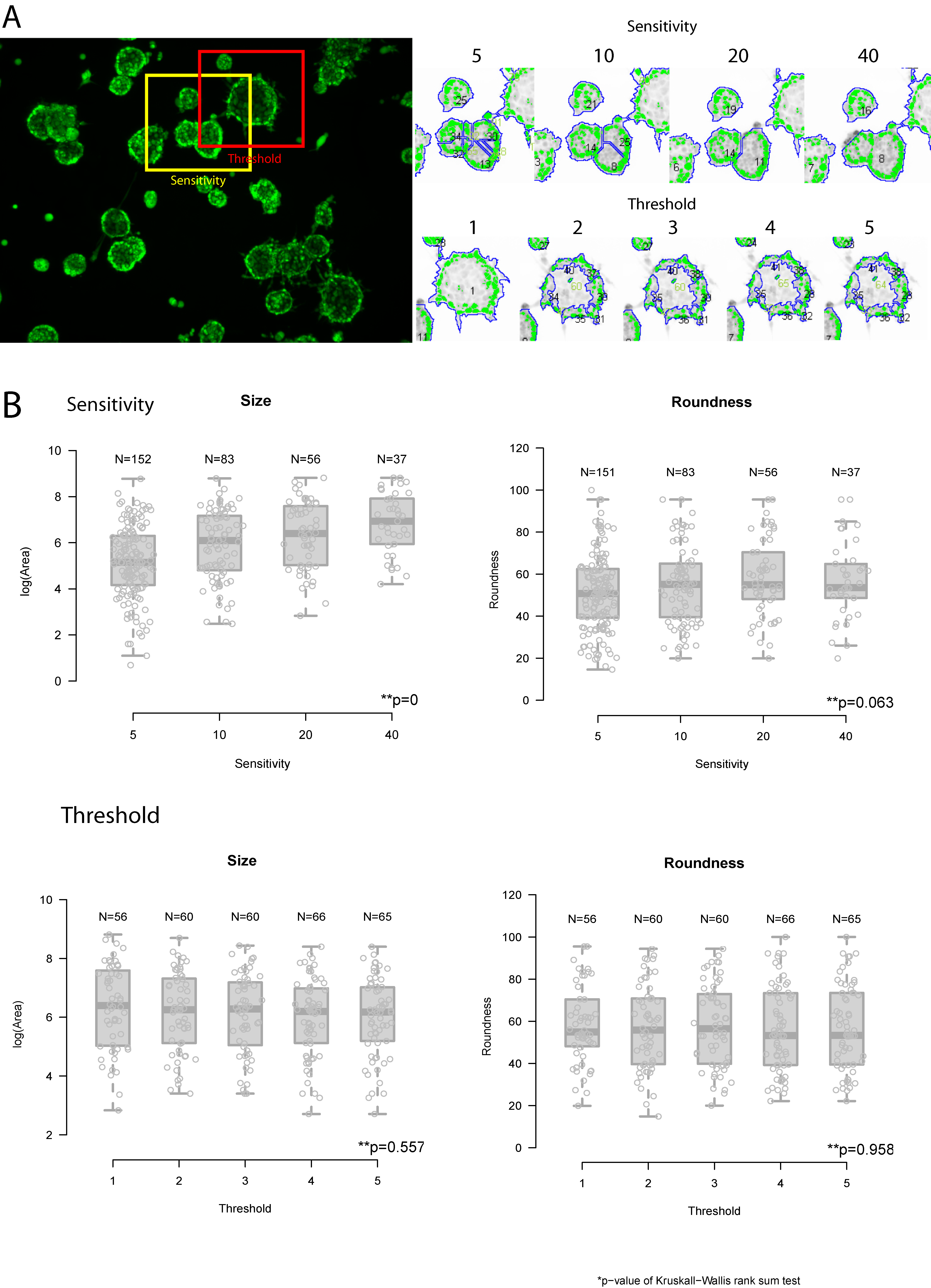

Supplement: Figure S1 — Impact of modifying the AMIDA program parameters “sensitivity” and “threshold” on segmentation. (A) An image of PC-3 cells cultured 10 days in 3D was used as an example (A: left image). Modifying “sensitivity” from values of 5 to 40 (with constant threshold 1) results in reduced fragmentation of adjacent spheroid structures (A: upper right panel). The “threshold” parameter has opposite effect: increasing the value from 1 to 5 (with constant sensitivity at 20) has a notable effect on fragmentation (A: lower right panel). (B) The effect of modifying “sensitivity” and “threshold” parameters was statistically evaluated by Kruskal-Willis rank sum test. As expected, increasing the “sensitivity” value yields larger (p = 0) and fewer cellular structures (sensitivity 5: N = 152, sensitivity 40: N = 37). However, symmetry (Roundness) is not significantly affected. A higher threshold value tends to identify more structures (threshold 1: N = 56, threshold 5: N = 65) but has no significant effect on structure size or symmetry measures in this case. (TIF) [file pone.0096426.s001.tif]

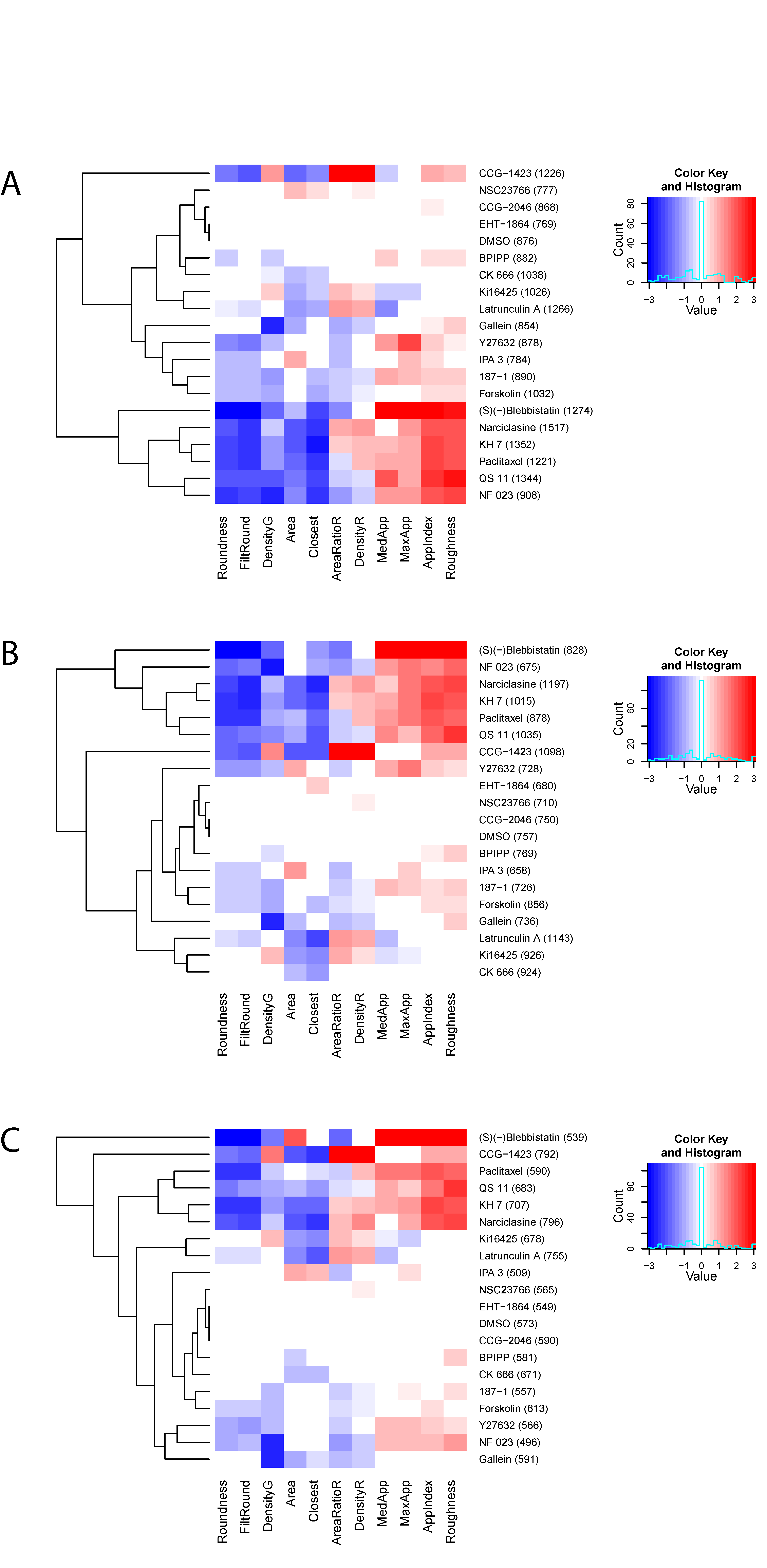

Supplement: Figure S2 — Impact of modifying the AMIDA “sensitivity” parameter on a whole experiment level. Non-invasive DU145 cells/spheroids were cultured 4 days in 3D Matrigel matrix and exposed to 19 different compounds for 6 days. The 3D cell cultures were imaged with spinning disk confocal microscope and the maximum intensity projection images were analysed using three different sensitivity settings at (A) = 10, (B) = 20 and (C) = 40 (threshold: constant setting at t = 1, size >100 pixels). The heatmaps show the standardized, p-value filtered (Bonferroni-corrected Mann-Whitney U-test p<0.05) differences in medians between treatments and DMSO controls for the selected features. Both the treatments and the morphological parameters are hierarchically clustered based on complete linkage of Euclidean distances, enabling unbiased evaluation. The total number of observations ( = spheroids) for each treatment is indicated in parentheses. “Sensitivity” values of 20 and 40 yield almost identical clusters, whereas the value 10 stands out as clearly different, most probably because of heavier fragmentation. (TIF) [file pone.0096426.s002.tif]

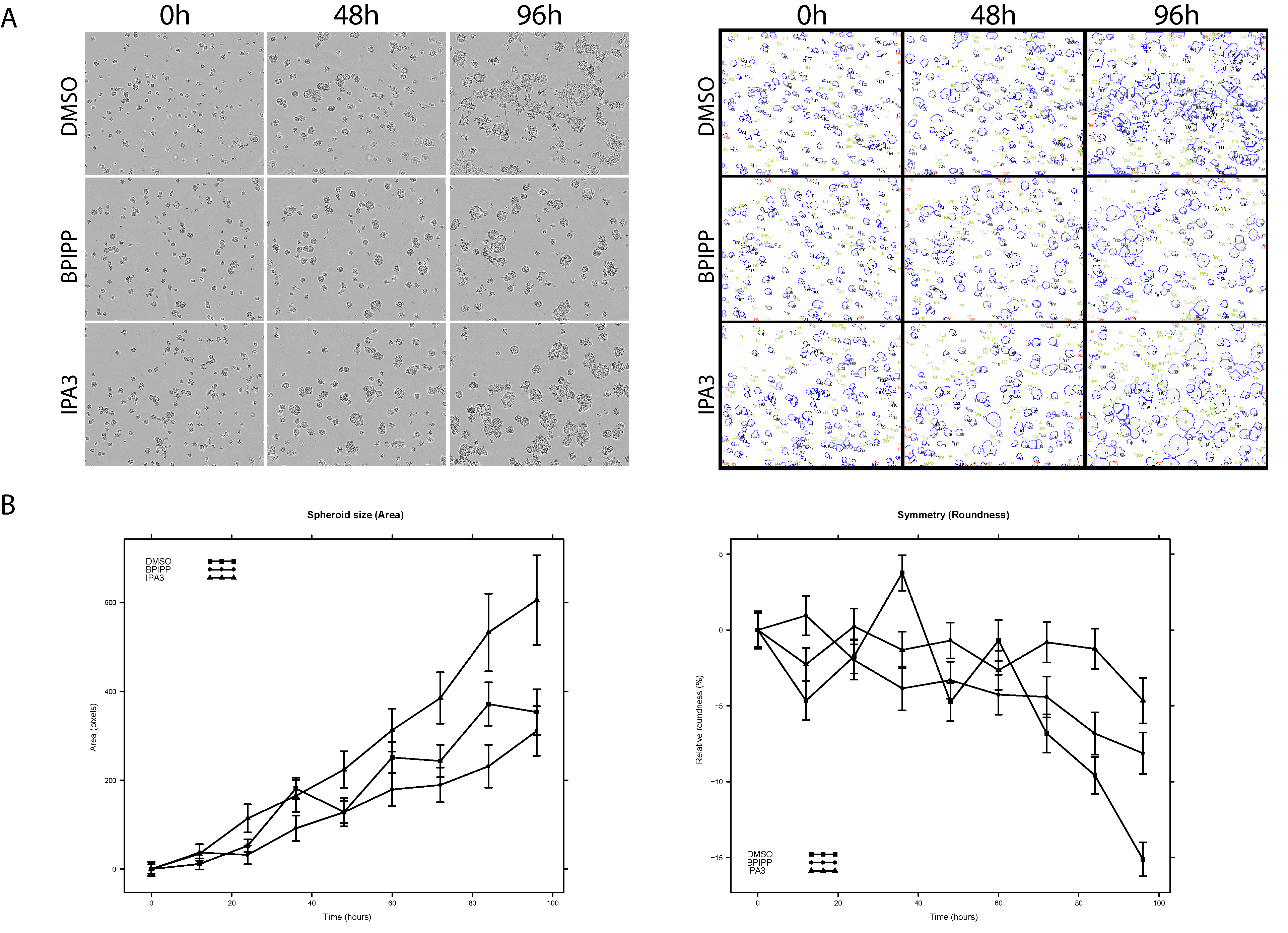

Supplement: Figure S3 — Exemplary evaluation of segmentation and image analysis of phase contrast images, using AMIDA. (A) Original phase contrast images as derived from IncuCyte (left), and after background subtraction and segmentation (right). (B) Time course of spheroid growth (left graph) for control (DMSO) compared to two compound treatments (BPIPP and IPA3) known to primarily affect tumor cell invasiveness. With DMSO, most spheroids undergo invasive transformation after 100 h of treatment, which is partly inhibited by BPIPP and IPA3 (right graph). (TIF) [file pone.0096426.s003.tif]

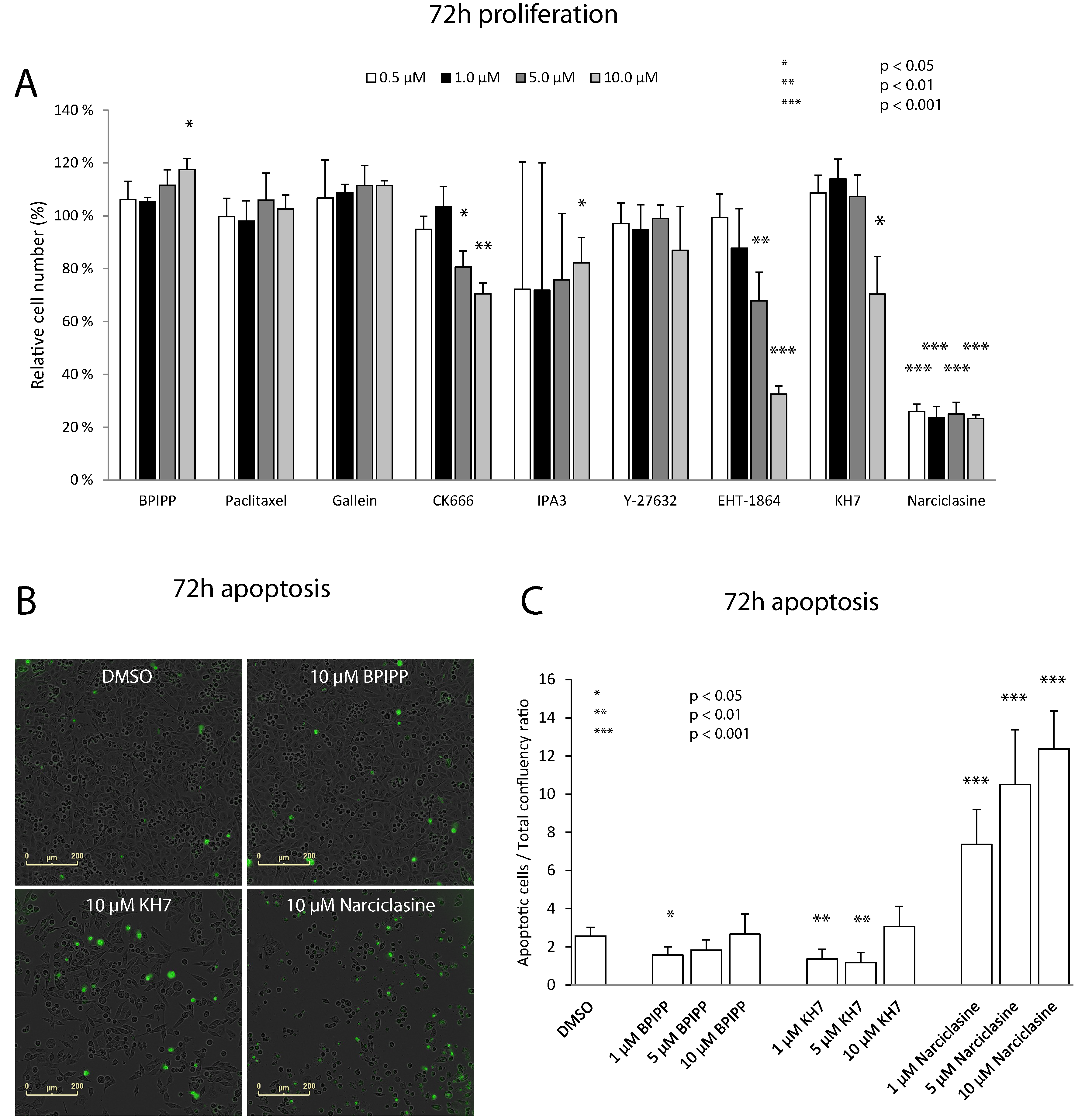

Supplement: Figure S4 — Validation of dynamic responses observed in 3D culture, using standard 2D monolayer assays. (A) Proliferation: PC-3 cells were treated for 72 h with 4 concentrations of each compound. Cell numbers were assessed by nuclear staining with Hoechst (results shown as percentage of the DMSO control, 204–1841 nuclei counted per treatment). (B) Apoptosis: PC3 cells were treated in 2D monolayer with three compounds that induce apoptosis in 3D settings, namely adenylate-cyclase inhibitors BPIPP and KH7, and RhoA activator narciclasine, and stained with NucView 488 caspase-3 substrate to detect apoptotic nuclei. (C) Apoptosis was quantified from 2D image data using IncuCyte (2011A Rev2) object counting tool (v2.0). The quantification indicates that narciclasine massively induces programmed cell death, while all other drugs only result in small increases of apoptosis at the highest (10 µM) concentrations. (TIF) [file pone.0096426.s004.tif]

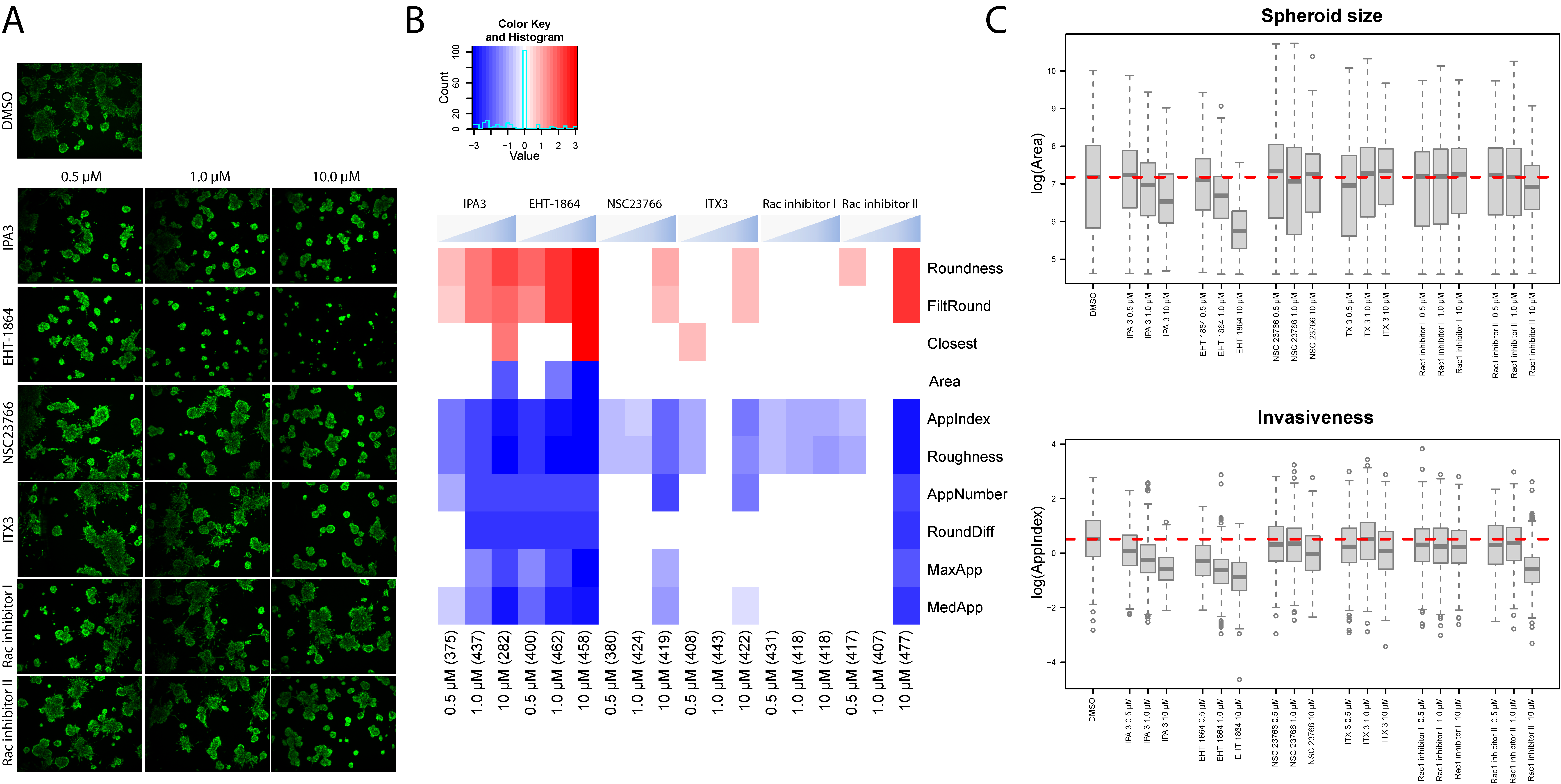

Supplement: Figure S5 — Evaluation of anti-invasive effects of several Rac-related inhibitors on PC-3 cells cultured in 3D Matrigel matrix for 10 days. (A) Spinning disk confocal microscope (5x objective) image projections of PC-3 spheroids exposed to six inhibitors – namely IPA3 (Group I p21-activated kinase or PAK inhibitor), EHT-184 (non-selective Rac family GTPase inhibitor), NSC23766 (selective Rac1-GEF inhibitor), ITX3 (selective TrioN RhoGEF inhibitor), Rac inhibitor I (Merck #553502) and Rac inhibitor II (Merck #553511) – all in three concentrations (0.5, 1 and 10 µM) for six days (days 4-10), stained at day 10 with calcein AM live cell colour. (B) A heatmap of AMIDA generated morphometric data displaying p-value filtered (Mann-Whitney U-test, Bonferroni-corrected cut-off p<0.05) standardized median differences across 10 selected morphological features. (C) Boxplots highlighting clear dose-responses for spheroid size and invasiveness in response to several Rac-related inhibitors, most notably IPA3, EHT-1864, NSC23766, ITX3 and Rac inhibitor II. (TIF) [file pone.0096426.s005.tif]

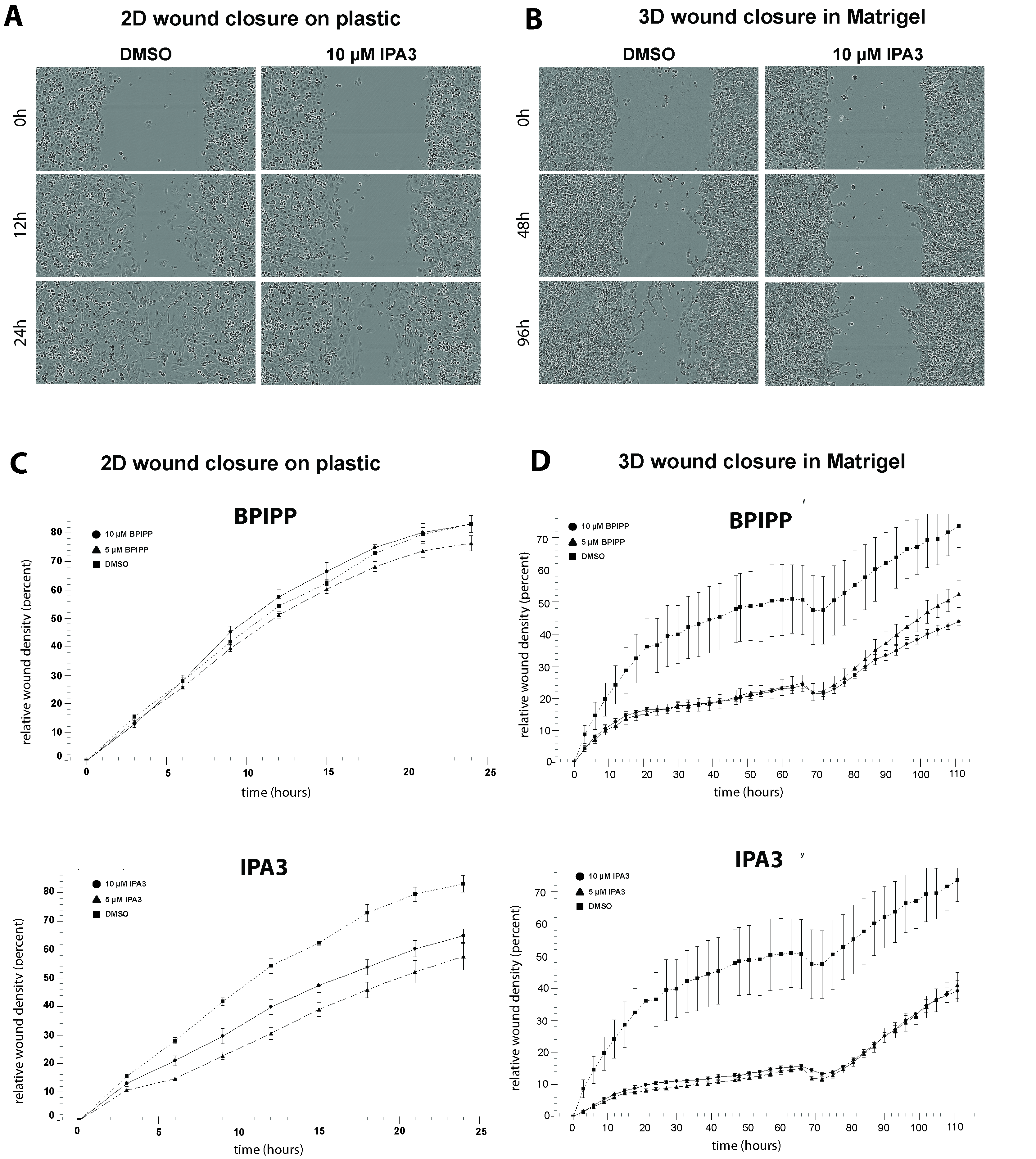

Supplement: Figure S6 — Validation of altered cell migration and motility measured in 2D and 3D, using PC3 cells. (A) 2D Scratch wound migration and (B) 3D invasion assays in Matrigel, treated with the IPA3 compound. (C and D) Quantification of cell motility in 2D cultures using IncuCyte (2010A Rev2), treated with compounds that were most specifically active invasion suppressors in 3D: adenylate-cyclase inhibitor BPIPP and PAK-class I inhibitor IPA3. Compounds were administered in two different concentrations. (C) In the 2D migration assays, a confluent PC-3 monolayer cultured on Essen ImageLock plates was wounded with Essen CellPlayer, wound closure monitored for 24 h, and quantified by IncuCyte imaging. The wound closure was measured as wound cell density in relation to the original wound area. (D) In 3D invasion assays, confluent cell layers were scratched on Matrigel-coated ImageLock plates and covered by an additional layer of Matrigel, containing the compounds. Wound closure was monitored for 112 h, and quantified with IncuCyte. Time series illustrating delayed wound closure in response to IPA3, a PAK1 inhibitor, both in 2D migration and invasion assays. (TIF) [file pone.0096426.s006.tif]

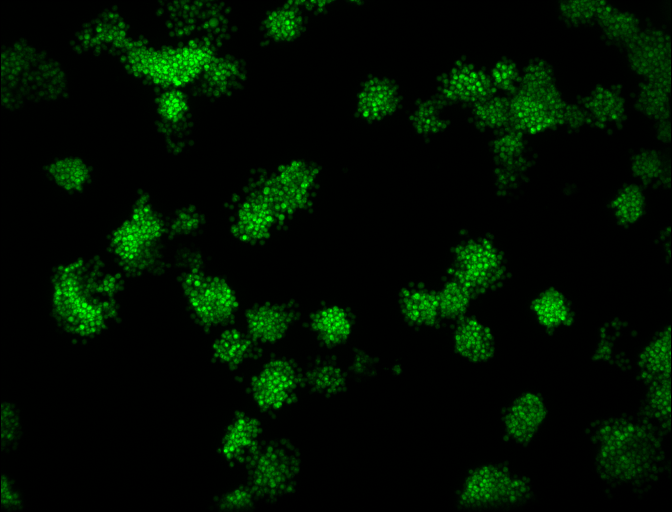

Supplement: Image Data S1 — Compressed ZIP file contains a set of exemplary test images derived from 3D cultures of HeLa and PC3 cells, in different formats and resolutions. These images can be analysed with the AMIDA software. (ZIP) [file pone.0096426.s011.zip › HeLaGFP - Slide 01 - Position 15.tif]

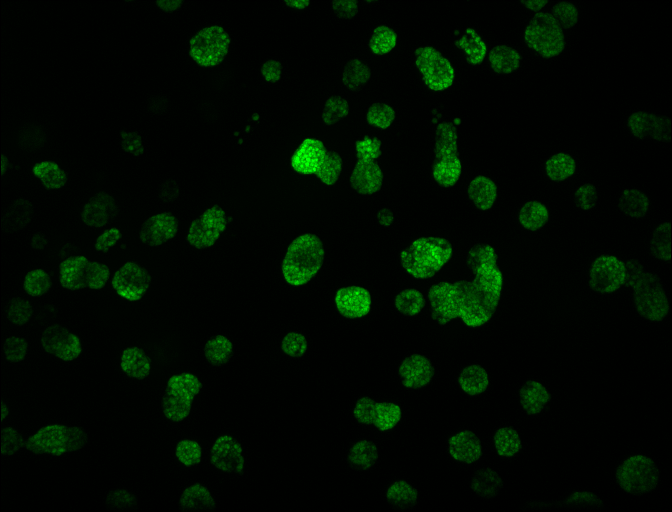

Supplement: Image Data S1 — Compressed ZIP file contains a set of exemplary test images derived from 3D cultures of HeLa and PC3 cells, in different formats and resolutions. These images can be analysed with the AMIDA software. (ZIP) [file pone.0096426.s011.zip › HeLaGFP - Slide 01 - Position 16.tif]

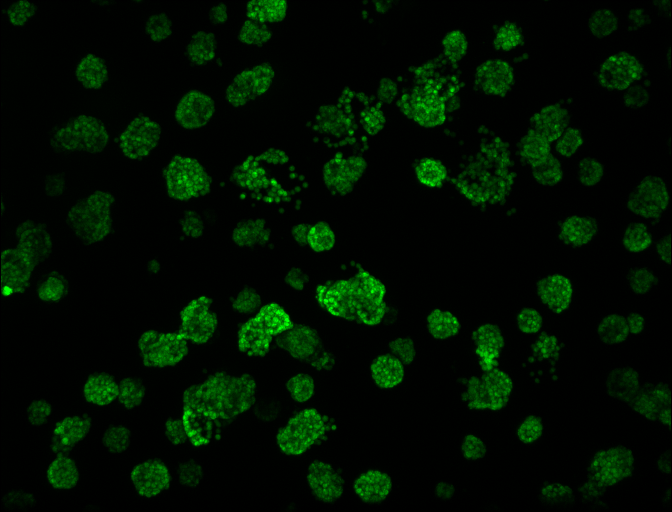

Supplement: Image Data S1 — Compressed ZIP file contains a set of exemplary test images derived from 3D cultures of HeLa and PC3 cells, in different formats and resolutions. These images can be analysed with the AMIDA software. (ZIP) [file pone.0096426.s011.zip › HeLaGFP - Slide 01 - Position 17.tif]

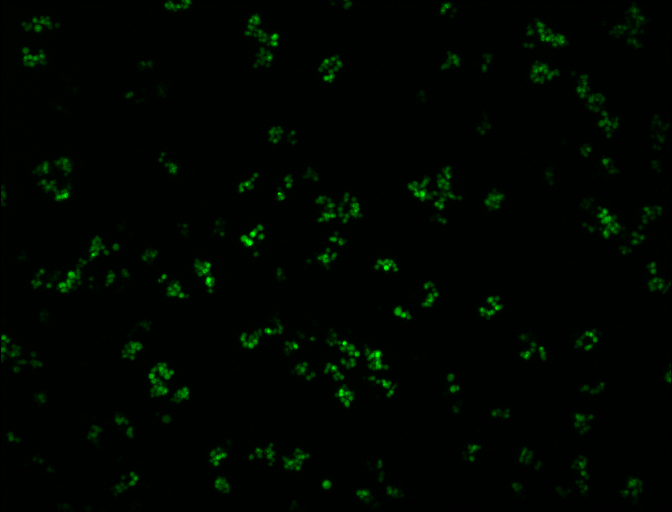

Supplement: Image Data S1 — Compressed ZIP file contains a set of exemplary test images derived from 3D cultures of HeLa and PC3 cells, in different formats and resolutions. These images can be analysed with the AMIDA software. (ZIP) [file pone.0096426.s011.zip › HeLaGFP - Slide 01 - Position 30.tif]

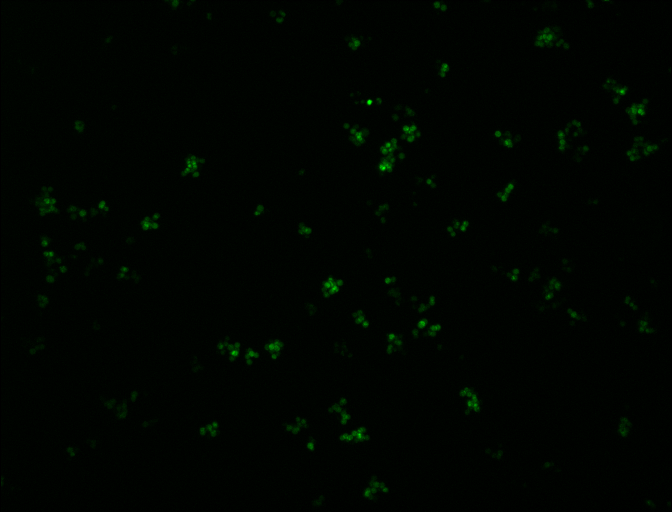

Supplement: Image Data S1 — Compressed ZIP file contains a set of exemplary test images derived from 3D cultures of HeLa and PC3 cells, in different formats and resolutions. These images can be analysed with the AMIDA software. (ZIP) [file pone.0096426.s011.zip › HeLaGFP - Slide 01 - Position 37.tif]

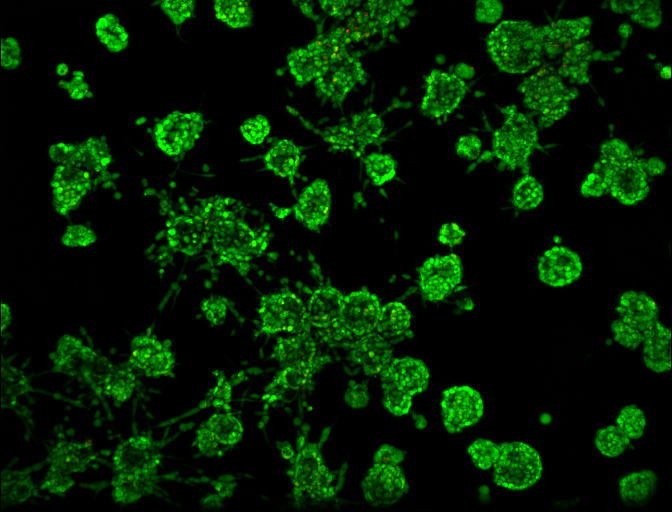

Supplement: Image Data S1 — Compressed ZIP file contains a set of exemplary test images derived from 3D cultures of HeLa and PC3 cells, in different formats and resolutions. These images can be analysed with the AMIDA software. (ZIP) [file pone.0096426.s011.zip › PC3 - slide 01 - Position 2.tif]

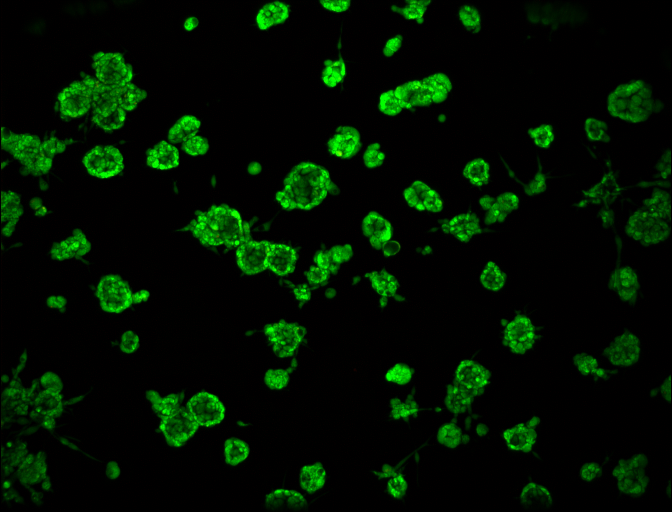

Supplement: Image Data S1 — Compressed ZIP file contains a set of exemplary test images derived from 3D cultures of HeLa and PC3 cells, in different formats and resolutions. These images can be analysed with the AMIDA software. (ZIP) [file pone.0096426.s011.zip › PC3 - slide 02 - Position 45.tif]

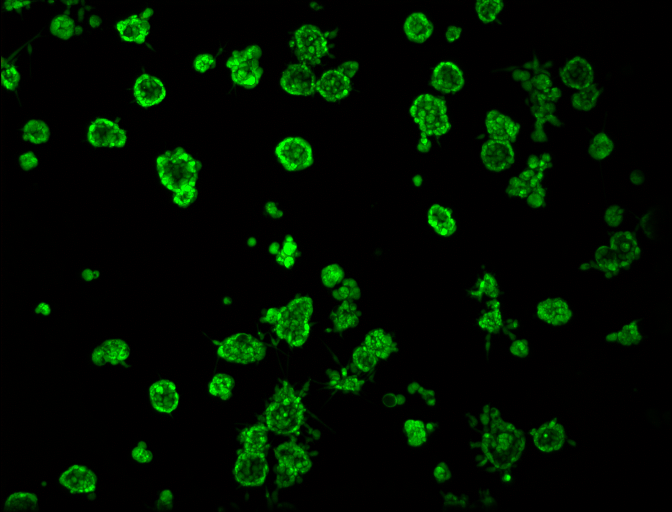

Supplement: Image Data S1 — Compressed ZIP file contains a set of exemplary test images derived from 3D cultures of HeLa and PC3 cells, in different formats and resolutions. These images can be analysed with the AMIDA software. (ZIP) [file pone.0096426.s011.zip › PC3 - slide 02 - Position 46.tif]

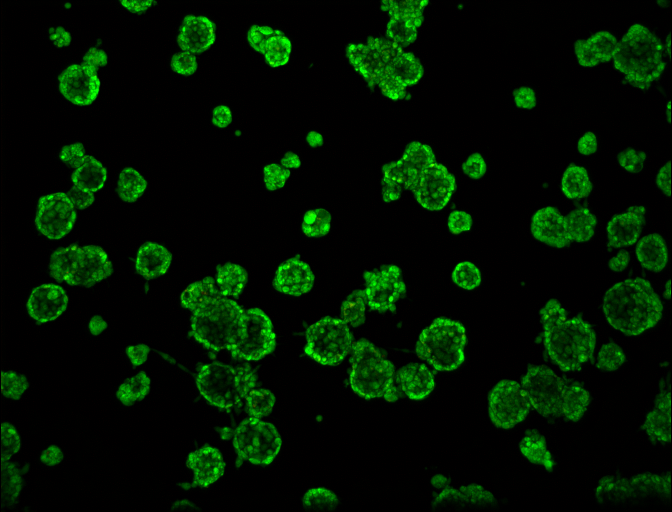

Supplement: Image Data S1 — Compressed ZIP file contains a set of exemplary test images derived from 3D cultures of HeLa and PC3 cells, in different formats and resolutions. These images can be analysed with the AMIDA software. (ZIP) [file pone.0096426.s011.zip › PC3 - slide 03 - Position 34.tif]

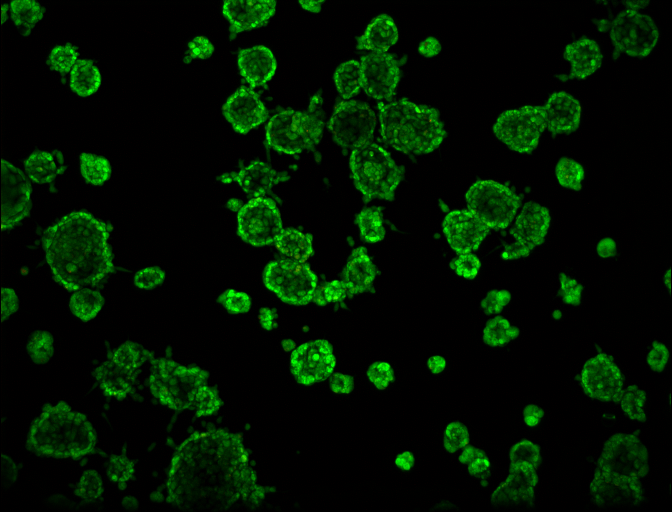

Supplement: Image Data S1 — Compressed ZIP file contains a set of exemplary test images derived from 3D cultures of HeLa and PC3 cells, in different formats and resolutions. These images can be analysed with the AMIDA software. (ZIP) [file pone.0096426.s011.zip › PC3 - slide 03 - Position 35.tif]

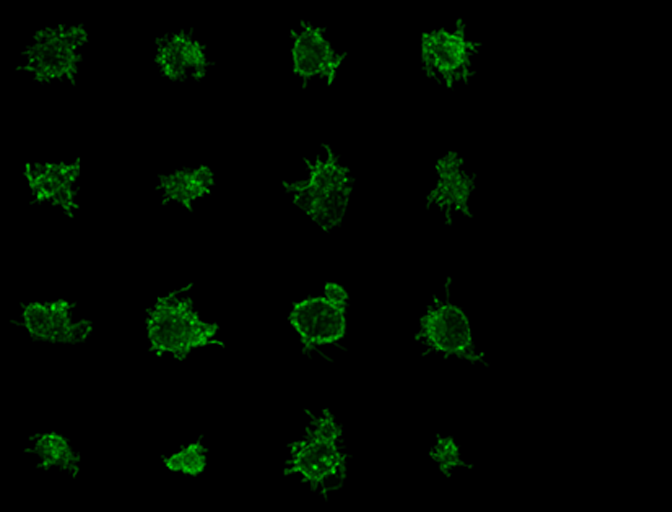

Supplement: Image Data S1 — Compressed ZIP file contains a set of exemplary test images derived from 3D cultures of HeLa and PC3 cells, in different formats and resolutions. These images can be analysed with the AMIDA software. (ZIP) [file pone.0096426.s011.zip › RGB - Slide 01 - position 01.tif]

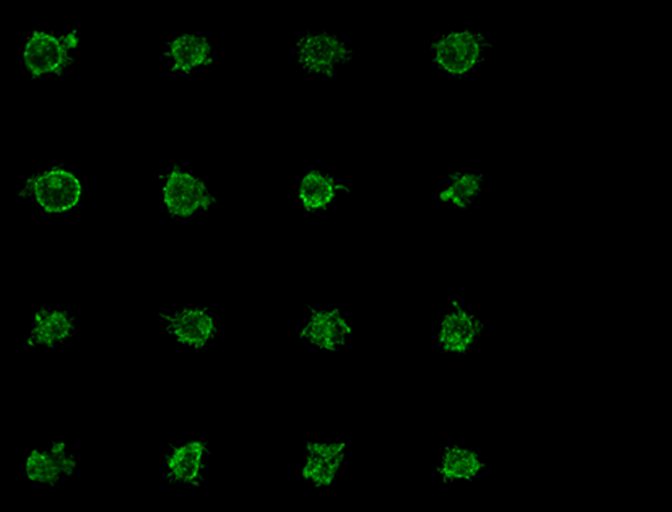

Supplement: Image Data S1 — Compressed ZIP file contains a set of exemplary test images derived from 3D cultures of HeLa and PC3 cells, in different formats and resolutions. These images can be analysed with the AMIDA software. (ZIP) [file pone.0096426.s011.zip › RGB - Slide 01 - position 02.tif]

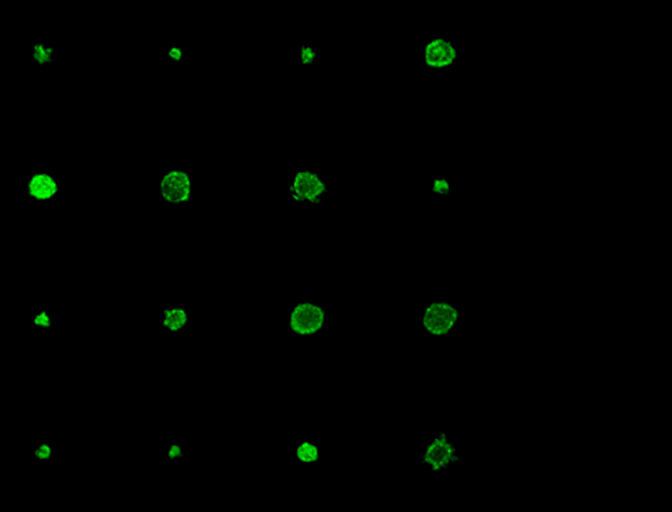

Supplement: Image Data S1 — Compressed ZIP file contains a set of exemplary test images derived from 3D cultures of HeLa and PC3 cells, in different formats and resolutions. These images can be analysed with the AMIDA software. (ZIP) [file pone.0096426.s011.zip › RGB - Slide 01 - position 03.tif]

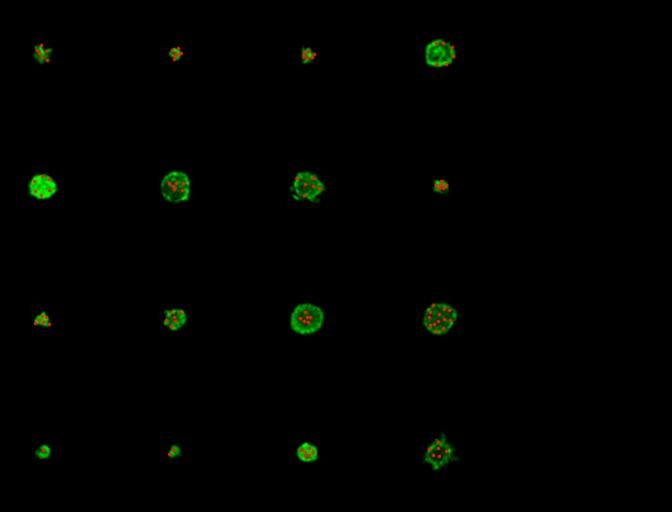

Supplement: Image Data S1 — Compressed ZIP file contains a set of exemplary test images derived from 3D cultures of HeLa and PC3 cells, in different formats and resolutions. These images can be analysed with the AMIDA software. (ZIP) [file pone.0096426.s011.zip › RGB - Slide 01 - position 04.tif]

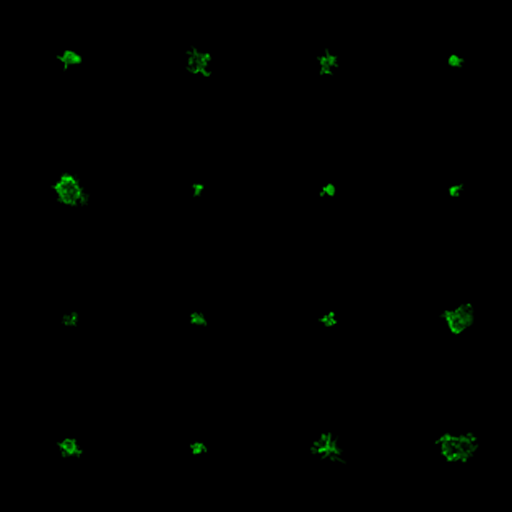

Supplement: Image Data S1 — Compressed ZIP file contains a set of exemplary test images derived from 3D cultures of HeLa and PC3 cells, in different formats and resolutions. These images can be analysed with the AMIDA software. (ZIP) [file pone.0096426.s011.zip › RGB - Slide 01 - position 05.tif]
