# Supplementary material for: Quantification of Dynamic Morphological Drug Responses in 3D Organotypic Cell Cultures by Automated Image Analysis
Source: PLoS One. 2014 May 8;9(5):e96426. doi: 10.1371/journal.pone.0096426 (PMC4014501; doi:10.1371/journal.pone.0096426)
Supplement: Table S1 — List of all cell lines used in the validation screens. (DOCX) [file pone.0096426.s007.docx]

**Supplemental Table 1.** List of cell lines used in validation screens.

| **Line** | **Origin** | **Details** | **Source** | **Reference** |
| --- | --- | --- | --- | --- |
| **EP156T** | Non-transformed prostate epithelial | Immortalized with pBabe-hTERT-puro retroviral vector | Varda Rotter (Weizmann Institute of Science, Rehovot, Israel) | Kogan I, et al., Cancer Res. 2006 Apr ;66(7):3531-40. |
| **RWPE-1** | Non-transformed prostate epithelial | Immortalized by human papilloma virus 18 (HPV-18) | ATCC (CRL-11609) | Bello D, et al., Carcinogenesis 18: 1215-1223, 1997. PMID 9214605 |
| **PC-3** | Prostate adenocarcinoma | Bone metastasis | ATCC (CRL-1435) | Kaighn ME, et al., Invest. Urol. 17: 16-23, 1979. PMID 447482 |
| **PC3-M pro4** | Prostate adenocarcinoma | Highly metastatic variant of PC3 | Isaiah Fidler | Clinical Cancer Research 1996 2; 1627 |
| **DU145** | Prostate adenocarcinoma | Brain metastasis | ATCC (HTB-81) | Stone KR, et al., Int. J. Cancer 21: 274-281, 1978. PMID 631930 |
| **LNCaP** | Prostate adenocarcinoma | Left supraclavicular lymph node metastasis | ATCC (CRL-1740) | Horoszewicz JS, et al. LNCaP model of human prostatic carcinoma. Cancer Res. 43: 1809-1818, 1983. PMID 6831420 |
| **22Rv1** | Prostate adenocarcinoma | Androgen independent line derived from CWR22 xenograft model | ATCC (CRL-2505) | Sramkoski RM, et al., In Vitro Cell. Dev. Biol. Anim. 35: 403-409, 1999. PMID 10462204 |
| **LAPC-4** | Prostate primary transitional cell carcinoma | Lymph node metastasis of xenografted mouse | Charles Sawyer (Univ. of California, Los Angeles, CA) | Klein KA, et al., Nat Med 1997;3:402–408. |
| **ALVA31** | Prostate adenocarcinoma | PC3 derivative | Department of Veterans Affairs Medical Center, Tacoma, WA | Mehta PP, et al., Mol Carcinog. 1996 Jan;15(1):18-32. |
| **MDA-MB-231** | Breast adenocarcinoma | Derived from metastatic site: pleural effusion | ATCC (HTB-26) | Cailleau R, et al. Breast tumor cell lines from pleural effusions. J. Natl. Cancer Inst. 53: 661-674, 1974. |
| **MDA-MB-231(SA)** | Breast adenocarcinoma | Spontaneous in vitro derivative of MDA-MB-231 cells. Highly metastatic in vivo. | Theresa Guise (Indiana University, Indianapolis, IN, USA) | Pollari S, et al. Breast Cancer Res Treat. 2011 Jan;125(2):421-30. doi: 10.1007/s10549-010-0848-5. |
| **HMEC** | Non-transformed primary mammary epithelial cells |  | Purchased from LONZA |  |
