# Supplementary material for: Quantification of Dynamic Morphological Drug Responses in 3D Organotypic Cell Cultures by Automated Image Analysis
Source: PLoS One. 2014 May 8;9(5):e96426. doi: 10.1371/journal.pone.0096426 (PMC4014501; doi:10.1371/journal.pone.0096426)
Supplement: Table S2 — Estimated standard deviation parameter values for random effects. The values are shown for three morphological parameters, logarithmic Area, Roundness and logarithmic. (DOCX) [file pone.0096426.s008.docx]

**Supplemental Table 2.** Estimated standard deviation parameter values for random effects. The values are shown for three morphological parameters, logarithmic Area, Roundness and logarithmic AppIndex, and for a selection of prostate and breast cancer cell lines.

| logArea |  |  |  |
| --- | --- | --- | --- |
| **Cell line** | **Between positions** | **Between wells** | **Residual** |
| ALVA31 | 0 | 0.07 | 1.08 |
| DU145 | 0.04 | 0 | 0.72 |
| MDA-MB-231(SA) | 0.14 | 0.08 | 1.09 |
| MDA-MB-231 | 0.06 | 0.03 | 0.75 |
| RWPE-1 | 0.05 | 0.08 | 0.85 |
| PC-3 | 0.14 | 0.08 | 0.87 |
| PC-3M Pro 4 | 0.11 | 0.04 | 0.99 |
| EP156T | 0.09 | 0.07 | 0.71 |
| LAPC-4 | 0.06 | 0 | 0.72 |
| LNCaP | 0.09 | 0.07 | 0.75 |
| Roundness |  |  |  |
| **Cell line** | **Between positions** | **Between wells** | **Residual** |
| ALVA31 | 0.79 | 0.46 | 15.99 |
| DU145 | 0.89 | 1.06 | 15.03 |
| MDA-MB-231(SA) | 3.11 | 0 | 17.13 |
| MDA-MB-231 | 0.98 | 0 | 18.22 |
| RWPE-1 | 1.28 | 0.97 | 14.7 |
| PC-3 | 1.39 | 0 | 15.22 |
| PC-3M Pro 4 | 2.15 | 0 | 18.13 |
| EP156T | 2.35 | 1.56 | 14.3 |
| LAPC-4 | 0.84 | 0 | 14.56 |
| LNCaP | 1.95 | 1.15 | 16.5 |

| logAppIndex |  |  |  |
| --- | --- | --- | --- |
| **Cell line** | **Between positions** | **Between wells** | **Residual** |
| ALVA31 | 0.07 | 0 | 0.81 |
| DU145 | 0.09 | 0.04 | 0.82 |
| MDA-MB-231(SA) | 0.12 | 0 | 0.75 |
| MDA-MB-231 | 0.05 | 0.06 | 0.92 |
| RWPE-1 | 0.09 | 0.04 | 0.65 |
| PC-3 | 0.09 | 0.06 | 0.7 |
| PC-3M Pro 4 | 0.13 | 0.04 | 0.9 |
| EP156T | 0.16 | 0.07 | 0.77 |
| LAPC-4 | 0.07 | 0.05 | 0.73 |
| LNCaP | 0.12 | 0 | 0.74 |
