# Supplementary material for: Quantification of Dynamic Morphological Drug Responses in 3D Organotypic Cell Cultures by Automated Image Analysis
Source: PLoS One. 2014 May 8;9(5):e96426. doi: 10.1371/journal.pone.0096426 (PMC4014501; doi:10.1371/journal.pone.0096426)
Supplement: Table S3 — A summary of pseudo codes used in AMIDA. (DOCX) [file pone.0096426.s009.docx]

**Supplemental Table 3.** AMIDA pseudo codes.

| 1. Initial threshold selection |
| --- |
| **** |
| 1. Amida pseudo code |
| **** |
